# An integrated subtractive genomics and immunoinformatics approach for designing a universal multi-epitope vaccine against *Brucella* spp.

**DOI:** 10.3389/fbinf.2026.1818265

**Published:** 2026-07-07

**Authors:** Rhitam Biswas, Swapno Surabhi Sinha, Aditi Roy, Sudha Ramaiah, Anand Anbarasu

**Affiliations:** 1 Department of Biotechnology, School of Biosciences and Technology (SBST), Vellore Institute of Technology (VIT), Vellore, Tamil Nadu, India; 2 Department of Biosciences, School of Biosciences and Technology (SBST), Vellore Institute of Technology (VIT), Vellore, Tamil Nadu, India

**Keywords:** *Brucella* spp., cloning, molecular docking, molecular dynamics simulation, multi-epitope vaccine, pan-genome, reverse vaccinology

## Abstract

**Introduction:**

*Brucella* spp. are Gram-negative bacteria accountable for brucellosis in immunocompromised individuals and livestock. Due to the slow-growing latent phenotype, current antibiotics are insufficient to treat the infection. The lack of an approved vaccine for human use against this pathogen represents a significant public health concern and indicates the urgent need for novel prophylactic interventions.

**Methodology:**

In this study, the reverse vaccinology method was combined with pan-genome analysis to identify potential vaccine targets. Proteins have been screened for antigenicity, solubility, immunogenicity, and subcellular localization. B cell and T cell epitopes exhibiting high immunogenicity and solubility have been identified. Multi-epitope vaccine constructs have been evaluated and further analyzed depending on their physicochemical properties. Molecular docking, conformational dynamics, *in silico* cloning, and immune simulations were conducted to identify the optimal vaccine candidate.

**Results:**

Four proteins, trigger factor, outer membrane protein assembly factor BamA, urease subunit beta (UreB), and urease subunit alpha (UreC1) were considered for potential vaccine targets. A total of 26 B cell and 97 T cell epitopes with notable immunogenicity and solubility have been shortlisted. Twelve multi-epitope vaccine constructs were generated, among which Vc7 has been chosen based on structural and physicochemical properties. Molecular docking analysis revealed a good correlation with 2FSE and 2Z65, which were further analyzed to reveal that Vc7 exhibited stronger binding affinity (−135.24 kcal/mol) towards 2FSE, mediated by hydrophobic contacts, salt bridges, and intermolecular hydrogen bonds, making it the ideal vaccine complex and validated through a 150 ns molecular dynamics simulation. *In silico* cloning established construct compatibility, and immune simulation confirmed Vc7’s potential to elicit T cell, B cell, antibody, and cytokine-mediated responses.

**Conclusion:**

Vc7 has been identified as a structurally stable and highly immunogenic construct, suggesting its potential as a universal multi-epitope vaccine candidate for the prevention of brucellosis.

## 1 Introduction

*Brucella* spp. are Gram-negative, non-motile, asporogenous, strictly aerobic, facultative intracellular organisms belonging to the class *Alphaproteobacteria* and are the primary causative agents responsible for zoonotic brucellosis in humans and domestic animals (Vashishtha and Kumar, 2024). Morphologically, they are predominantly coccobacilli, occurring singly or less frequently in short chains, with diameters ranging from 0.5 to 0.7 µm and lengths of 0.5–1.5 µm, and possess an average genome size of approximately 3.2 Mbp (Seleem et al., 2008; Zhao and Zai, 2025). The genus *Brucella* comprises ten recognized species, each typically associated with specific animal hosts. Notably, *Brucella melitensis* (*B. melitensis*) primarily infects goats, whereas *Brucella abortus* (*B. abortus*) and *Brucella suis* (*B. suis*) predominantly infect cattle and pigs, respectively (McCullough, 1978). Among these, *B. melitensis* is prevalent and virulent, followed by *B. suis*, both of which cause severe infections in animals and humans due to their high pathogenicity.

Humans acquire infection mainly through direct exposure to infected animals or their contaminated bodily fluids, through consumption of unpasteurized dairy products, through inhalation of contaminated aerosols, and, more rarely, through breastfeeding or through sexual transmission (Mustafa et al., 2024). Brucellosis, also known as Mediterranean fever, undulant fever, Malta fever, or Gibraltar fever, is endemic in Central Asia, the Middle East, Mediterranean Europe, Central America, Africa, and India. The World Health Organization (WHO) has categorized brucellosis as one of the most overlooked zoonotic diseases worldwide, despite its strong influence on population health (Islam et al., 2025).

After becoming transmissible to humans, *Brucella* is phagocytosed and enters the bloodstream, resulting in bacteremia and subsequent spread to multiple organs, including the reticuloendothelial system, liver, spleen, kidneys, joints, heart, and reproductive organs. Clinical manifestations that usually follow an incubation period of 1–4 weeks include fever, arthralgia, malaise, headache, and excessive sweating (Bukhari, 2018). Epidemiological studies have estimated the prevalence of human brucellosis at 1.6–2.1 million cases per year, indicating that the disease is increasingly affecting global health (Khairullah et al., 2024). According to recent data, it has a seroprevalence of around 15.1% in India and is also especially prevalent in developing countries (Sadaqat, 2023).

Although there are brucellosis-specific antibodies that can be used to diagnose this disease, it has become a major health concern because of its chronic nature, intracellular nature, and recurrent relapse (Laine et al., 2023). Among standard treatment regimens, doxycycline with rifampin or streptomycin is recommended (Qureshi et al., 2023). The doxycycline-streptomycin regimen is associated with lower rates of therapeutic failure and relapse (typically less than 5%), whereas the doxycycline-rifampicin regimen is associated with higher relapse rate or treatment failure rate (typically up to 15%) (Rowaiye et al., 2022; Zhu et al., 2024). Nonetheless, the need for intramuscular streptomycin excludes its usefulness in resource-limited settings (Hashemi et al., 2012). Low antibiotic penetration and intracellular survival, slow growth, and *Brucella*’s ability to enter a latent state are among the factors that contribute greatly to disease recurrence and chronicity, which eventually lead to reduced treatment efficacy (Lang et al., 1993).

At present, there is no approved vaccine for the prevention of human brucellosis (Ariza et al., 2007). Nevertheless, in recent work, they have suggested creating multi-component or peptide-based vaccines by systematically screening the *Brucella* exoproteome and secretome to induce protective immune responses (Mode et al., 2022). The capacity of *Brucella* to establish long-term persistence within host cells presents a major obstacle to vaccine development, as an effective vaccine must prevent both acute disease and chronic infection (Vives-Soto et al., 2024).

The progress of whole-genome sequencing and immunoinformatics has offered hopeful approaches in the discovery of new vaccine targets (Vishnu et al., 2015; Basu et al., 2023). Nucleic acid-based and computationally driven technologies offer useful alternatives to conventional approaches to vaccine development, as they tend to elicit broad and long-lasting immunity (Elbehiry et al., 2023; Biswas et al., 2026). Through pan-genomic research, it is possible to categorize core, dispensable, and strain-specific genes across a series of *Brucella* genomes, thereby identifying conserved and indispensable antigens that provide universal protection (Swetha et al., 2022). Reverse vaccinology goes a step further by selecting antigens by computationally identifying conserved, immunogenic, non-allergenic proteins that lack homology with human proteins, using genomic and proteomic data (Vishnu et al., 2017). Predicting B and T cell epitopes that may trigger significant humoral and cellular responses is enabled by the following immunoinformatic investigations (Yang et al., 2016).

Although several vaccine strategies against *Brucella* spp. have been investigated, limitations such as incomplete protection, antigenic variability, and insufficient induction of long-term cellular immunity remain major challenges. Since *Brucella* is a facultative intracellular pathogen, effective protection requires the activation of both humoral and cell-mediated immune responses. In this context, multi-epitope vaccines offer an advantage by incorporating multiple immunodominant B cell and T cell epitopes into a single construct, thereby enabling broad and targeted immune stimulation while reducing the presence of unwanted antigenic components (Moradkasani et al., 2025). Furthermore, integrating pan-genome analysis with reverse vaccinology and immunoinformatics offers an important advantage over conventional approaches by enabling the identification of highly conserved core antigens across multiple *Brucella* species and the rational selection of highly immunogenic, non-allergenic, and population-covering epitopes for universal vaccine design (Wang et al., 2022). Molecular docking, followed by molecular dynamics (MD) and immune simulation, is used to analyze the interactions of these identified epitopes with immune receptors, such as major histocompatibility complex (MHC) molecules and Toll-like receptors (TLRs), in a multi-epitope vaccine design. These epitopes are incorporated into a single construct with appropriate adjuvants and linkers (Sanchez-Trincado et al., 2017). Combinations of genomics, bioinformatics, structural biology, and computational immunology are effective in improving the accuracy of vaccine and drug discovery programs and accelerating the development of efficient therapies specific to brucellosis (Pradeepkiran et al., 2015; Olotu and Soliman, 2021).

**FIGURE 1:**
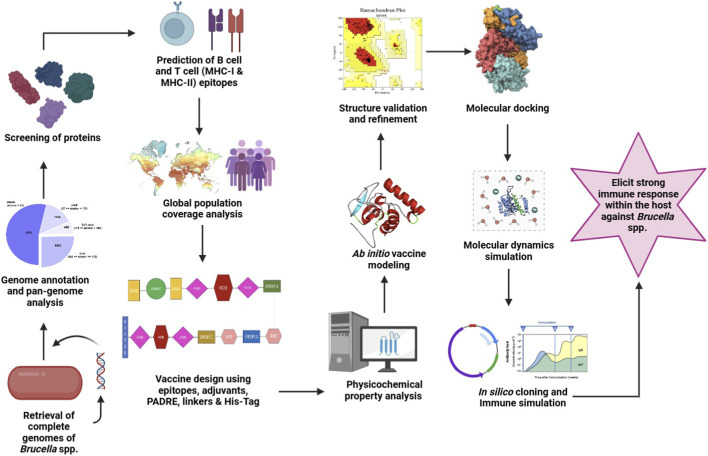
Schematic representation of the workflow used for the design and validation of a multi-epitope vaccine against *Brucella* spp.

In the present work, a comparative pan-genome analysis was performed to identify conservation of core protein targets across *Brucella* spp., followed by immunoinformatics analysis to aid the selection of a universal and unbiased vaccine candidate. In contrast to previous studies, which mainly used composite reverse vaccinology pipelines by relying on single-strain antigen selection or a limited subset of proteins (Zai et al., 2021; Yin et al., 2023; Nora and Khadidja, 2024; Shi et al., 2024), this investigation combines pan-genomics with reverse vaccinology to identify universally conserved and immunogenic targets for rational multi-epitope vaccine design against *Brucella* spp. Although a previous study performed pan-genome analysis of *Brucella* spp., it was limited to antigen presentation and subcellular localization assessment and did not advance to multi-epitope vaccine construction or structural validation (Hisham and Ashhab, 2018). In this study, candidate epitopes derived were screened using rigorous computational methods to assess antigenicity, allergenicity, immunogenicity, and solubility. Subsequently, various epitope combinations were systematically assembled into distinct multi-epitope constructs and comparatively analyzed for their predicted immunogenic potential, physicochemical properties, secondary- and tertiary-structure quality, structural stability, and receptor-binding affinity. These integrated immunological and structural assessments enabled rationale prioritization of the most promising vaccine construct, as shown in Figure 1.

## 2 Materials and methods

### 2.1 Genome retrieval and pan-genome annotation

A dataset of 1,729 *Brucella* spp. genomes was retrieved from the NCBI Genome database. To ensure high-quality comparative pan-genome analysis, only complete and fully assembled genomes were selected, while draft, fragmented, low-coverage, duplicate, and incomplete assemblies were excluded. Based on these selection criteria, 185 complete genomes representing diverse *Brucella* spp., including *B. melitensis*, *B. abortus*, *B. suis*, *B. canis*, and other representative species, were shortlisted for downstream pan-genome analysis. The selected genomes were downloaded in FASTA format and reannotated with Prokka v1.14.6, employing the Prodigal algorithm to ensure consistent gene prediction and annotation across all genomes (Seemann, 2014). Subsequently, the annotated genome files were analyzed using Roary v3.13.0 to profile the pan-genome and identify conserved core genes. It uses PRANK v170427, a stochastic multiple-sequence alignment program, to align core genes and generate a presence-absence matrix of genes (Page et al., 2015).

### 2.2 Prediction of novel protein targets

To minimize the possibility of autoimmune cross-reactivity, homologous proteins showing significant similarity to the human proteome were excluded through BLASTp analysis against the human reference proteome (Altschul et al., 1990). Proteins exhibiting sequence identity greater than 56%, substantial query coverage (≥50%), and statistically significant similarity with an E-value threshold of ≤0.005 were considered potentially cross-reactive and were therefore excluded from further analysis. The selected 56% identity threshold was chosen based on preliminary assessment of different homology-filtering criteria and their impact on candidate protein retention. More stringent thresholds substantially reduced the number of conserved *Brucella* proteins available for downstream analysis, whereas less stringent thresholds increased the retention of proteins exhibiting similarity to the human proteome. Therefore, 56% was selected as a balanced filtering threshold, enabling the identification of pathogen-specific vaccine candidates while minimizing potential host cross-reactivity. The stringent filtering criteria were employed to ensure the selection of pathogen-specific proteins while minimizing the risk of off-target immune responses and autoimmune reactions in the host. However, this filtering may potentially eliminate certain conserved bacterial proteins; only proteins with substantial similarity to the human proteome were removed. Furthermore, subsequent screening based on pan-genome conservation, subcellular localization, antigenicity, and solubility ensured the retention of biologically relevant, immunologically significant vaccine candidates for downstream analyses. The sequences were evaluated for subcellular localization using the Gneg-Ploc v2.0, CELLO, and PSORTb servers (Chou and Shen, 2006; Chou and Shen, 2008; Yu et al., 2006; Yu et al., 2010). The proteins screened were further tested for solubility and antigenic potential. Antigenicity was determined using two web servers, VaxiJen v2.0 and ANTIGENpro, which predict antigenicity based on protein microarray data (Sharma et al., 2023). These two tools utilize alignment-independent prediction, and a cut-off value of 0.3 was used to eliminate all but putative antigens (Magnan et al., 2010). SOLpro, a sequence-based filtration tool that predicts solubility on expression in *E. coli* (*Escherichia coli*), was further used to filter candidate proteins with a cut-off of 0.5 (Magnan et al., 2009). The proteins that met the antigenicity and solubility requirements were to be further analyzed.

### 2.3 Epitope prediction

The selected proteins have been further evaluated to identify potential T cell and B cell epitopes that could elicit a strong immune response.

#### 2.3.1 Class-I MHC epitope profiling

The ProPred-I server, IEDB MHC-I Binding Predictions, and IEDB Class I immunogenicity tools were used to identify MHC-I binding sites for the identified antigenic proteins. Potential MHC-I-binding regions and proteasomal cleavage sites in 46 MHC-I alleles were identified using ProPred-I, based on scoring matrices derived from the BIMAS database (Singh and Raghava, 2003). Both proteasome and immunoproteasome filters were set to a 3% threshold to cover a broad spectrum of HLA alleles. The IEDB MHC-I Binding Predictions integrates algorithms such as the Stabilized Matrix Method (SMM), Artificial Neural Network (ANN), and Average Relative Binding (ARB). Among them, ANN is the most precise technique for predicting the binding affinity of 9-mer peptides to 27 frequently occurring HLA alleles, compared with other methods (Peters et al., 2006). Peptides with IC50 values below 50 nM were considered strong binders and were therefore selected for further analysis. These 9-mer peptides were shortlisted and evaluated using the IEDB Class I Immunogenicity platform, which computes immunogenicity scores based on amino acid composition and position (Calis et al., 2013). Peptides having a score >0.15 were considered to possess strong immunogenic potential. To further evaluate their suitability as vaccine candidates, the selected peptides were analyzed using ToxinPred to assess potential toxicity and VaxiJen v2.0 to determine antigenic properties. This additional screening step helped ensure that the shortlisted peptides were both non-toxic and sufficiently antigenic, thereby supporting their safety and effectiveness for vaccine development. ToxinPred uses a Support Vector Machine (SVM)-based classifier and a 0.5 threshold to identify toxic epitopes, whereas VaxiJen uses a 0.4 threshold value to assess antigenicity based on physicochemical properties (Gupta et al., 2013). The final epitope candidates used to develop a vaccine were only those peptides that were strongly immunogenic, highly antigenic, and non-toxic.

#### 2.3.2 Class-II MHC epitope profiling

The antigenic peptides’ binding sites with MHC-II molecules were recognized with the help of the IEDB MHC-II Binding Predictions tool. The Consensus 2.22 algorithm was chosen among existing algorithms for its strength and broad applicability. IC50 values <50 nM and percentile rank <0.2 were used to characterize peptides that strongly interact with the HLA reference alleles (Biswas et al., 2024). These high-affinity peptides were further tested using the IEDB CD4 T cell Immunogenicity Prediction platform, which follows a recommended protocol to generate immunogenicity scores and a panel of seven standard HLA class II alleles to identify putative MHC-II epitopes (Paul et al., 2015). Peptides with scores below the 66% threshold were considered immunogenic. Shortlisted candidates were also screened for toxicity and antigenicity using ToxinPred and VaxiJen v2.0, respectively, to ensure they were safe and immunogenic. Finally, peptides that were non-toxic, highly antigenic, and immunogenic were retained for incorporation into the final vaccine construct.

#### 2.3.3 Comprehensive MHC clustering analysis

Specific binding of class-I and class-II MHC epitopes was further evaluated using the MHCcluster v2.0 tool to assess binding to individual MHC alleles. This program provides an overall picture of the functional relationships between HLA alleles and the selected epitopes and produces a heat map and a dendrogram, thereby increasing the probability of wide population coverage (Thomsen et al., 2013; Mortazavi et al., 2024). This analysis is essential for optimizing epitope selection and for including well-characterized epitopes with high interaction in the construction of an effective multi-epitope vaccine.

#### 2.3.4 Global HLA population distribution analysis

Population coverage of highly immunogenic T cell epitopes was evaluated utilizing the IEDB Population Coverage tool. It has been used to enter core epitope sequences and matching HLA alleles identified by both epitope-binding prediction platforms (Dhanda et al., 2019). Based on MHC restriction profiles and HLA genotypic frequencies, the program estimates the percentage of the target population that would react to the epitope set. It uses data on HLA allelic frequencies and MHC-binding limitations to estimate how many individuals worldwide are likely to mount an immune response to the epitopes. The analysis considers HLA allele distribution worldwide across more than 115 countries and 20 ethnic groups, divided into 16 geographical regions, and also enables the inclusion of custom parameters with user-specified allele frequencies (Swetha et al., 2016).

#### 2.3.5 B cell epitope profiling

Epitope recognition by B cells determines the humoral immune response. The BepiPred v3.0 and ABCPred web servers have been used to identify potential linear B cell epitopes. BepiPred forecasts epitope possibilities using a random forest algorithm, predicting the potential epitopes using antigen-antibody interaction patterns based on the fixed-length amino acid sequence of a protein, and ABCPred is an ANN that predicts epitope motifs (Saha and Raghava, 2006; Clifford et al., 2022). Both tools used default parameters, with a threshold of 0.5. These shared epitopes between the two servers were selected and analyzed.

#### 2.3.6 Epitope conservancy analysis

The conservancy of the final shortlisted epitopes incorporated into the vaccine construct was evaluated using the IEDB Epitope Conservancy Analysis Tool. Conservancy analysis was performed against homologous protein sequences retrieved from representative *Brucella* spp., including *B. melitensis*, *B. abortus*, *B. suis*, *B. canis*, and *B. ovis*. Each epitope was analyzed against the homologous sequences of its parent protein to assess the extent of sequence conservation across species. An identity threshold of 80% was employed to assess epitope conservancy. Epitopes exhibiting high conservancy were considered suitable candidates for inclusion in the universal multi-epitope vaccine construct.

### 2.4 Epitope selection and vaccine design

The predicted epitopes were prioritized through a sequential filtering strategy based on immunological and physicochemical criteria. Initially, B cell and T cell epitopes derived from the shortlisted proteins were screened for binding affinity to MHC alleles using predefined IC50 and percentile-rank thresholds. The shortlisted epitopes were subsequently evaluated for immunogenicity, antigenicity, toxicity, and population coverage. Only epitopes predicted to be highly immunogenic, strongly antigenic, non-toxic, and capable of interacting with multiple HLA alleles were retained for downstream vaccine construction. For B cell epitopes, only the overlapping consensus epitopes identified by both the BepiPred and ABCPred servers were selected to improve prediction reliability, whereas for T cell epitopes, common epitopes identified across multiple prediction platforms that met the defined filtering criteria were prioritized. The final pool comprising MHC class-I, MHC class-II, and B cell epitopes was systematically assembled into multiple non-identical vaccine constructs using suitable adjuvants, PADRE sequence, and linker combinations, followed by comparative evaluation based on physicochemical properties, structural quality, antigenicity, allergenicity, solubility, and stability parameters to identify the most promising vaccine candidate. The vaccine constructs differed in epitope arrangement, adjuvant positioning, linker organization, and overall sequence composition, enabling comparative assessment of their effects on structural and immunological properties. This strategic design was aimed at improving antigen presentation, enhancing immunogenicity, and facilitating downstream purification during the manufacturing process (Roy et al., 2011).

### 2.5 Evaluation of the vaccine constructs

The Expasy ProtParam tool has been utilized to evaluate physicochemical characteristics of vaccine constructs (Gasteiger et al., 2005). It provides estimates of the instability index, molecular weight, and grand average of hydropathicity (GRAVY). To evaluate the safety and immunogenicity of the candidates in more detail, their allergenic and antigenic profiles were identified using AlgPred v2.0, AllerTop v2.1, Vaxijen v2.0, and ANTIGENPro. Moreover, the solubility of candidates after expression was predicted using SOLPro. AlgPred detects allergenic properties by applying several computational methods, including MERCI motif scanning, random forest classifiers, and BLAST-based similarity searches (Sharma et al., 2021). AllerTOP uses auto-cross-covariance (ACC) analysis, along with a k-nearest neighbors (kNN) algorithm, to predict allergenicity based on protein sequence transformations (Dimitrov et al., 2013). Collectively, these tests ensured that the vaccine constructs had appropriate physicochemical stability, solubility, and immunological characteristics.

### 2.6 Modeling of vaccine constructs and molecular docking

Multi-epitope vaccine constructs have been refined and structurally validated to analyze the final constructs.

#### 2.6.1 Modeling and structural validation of the vaccine constructs

3D geometry of vaccine constructs has been predicted using trRosetta, a deep learning-based protein structure prediction platform that uses multiple sequence alignments to predict inter-residue geometries (Anishchenko et al., 2021). These estimates are used to inform Rosetta-based energy minimization, thereby enabling accurate modeling of protein tertiary structures (Yang et al., 2020). The highest quality structural model among the generated models was selected. MolProbity, Procheck, and SAVES v6.0 were used to validate the chosen model. The quality of models has been assessed by calculating ideal geometric parameters, minimal steric clashes, and residues with more than 95% in preferred regions of the Ramachandran plot (Davis et al., 2007; Bagaria et al., 2012). The dynamics of every residue in the vaccine construct were measured using DynaMine, which employs NMR chemical shift data to predict residue-level flexibility as S^2^ order parameters. S^2^ values above 0.8 were considered rigid, and those below 0.8 were considered flexible regions affecting conformational adaptability (Cilia et al., 2014). The SOPMA server was used to recognize secondary structural elements, such as α-helices, β-sheets, and coils (Geourjon and Deléage, 1995). The construct that showed the best structural stability and the most appropriate functional suitability was selected for further study.

#### 2.6.2 Retrieval and optimization of the target proteins

The 3D structures of the target proteins were retrieved from the RCSB Protein Data Bank (PDB) using the following PDB IDs: 1H15, 2FSE, 1A6A, 2SEB, 3C5J, 2Q6W, and 2Z65. These HLA alleles were selected based on their availability as experimentally resolved crystal structures in the Protein Data Bank, their frequent utilization as representative reference alleles in immunoinformatics studies, and their broader relevance to global population coverage. TLR4 was selected owing to its established role in mediating innate immune recognition and activation against Gram-negative bacterial pathogens (Kim et al., 2023).

Swiss-PDBViewer (SPDBV) v4.1 was used as the initial preprocessing tool, including the removal of unwanted heteroatoms and the repair of missing residues. The refinement was then done by the GalaxyRefine server to minimize steric clashes, reduce Ramachandran outliers, and fix improper rotamers (Heo et al., 2013). Lastly, SPDBV was used to minimize the energy of refined protein models using the GROMOS96-43B1 force field, with 2000 steps of steepest-descent and conjugate-gradient methods (Guex and Peitsch, 1997).

#### 2.6.3 Conformational B Cell epitope mapping

The IEDB ElliPro program has been used to predict discontinuous B cell epitopes in the improved 3D structure of the vaccine construct. It determines conformational B cell epitopes on the protein surface by combining geometric properties with protrusion index calculations using an adapted Thornton approach. Moreover, the tool groups surface-accessible residues using a residue-based clustering algorithm, thereby identifying epitope-rich areas (Ponomarenko et al., 2008).

#### 2.6.4 Disulfide engineering

Disulfide engineering is the technique of adding or removing disulfide bonds to a protein’s structure to increase structural stability and rigidity. The sulfur atoms of cysteine residues are covalently linked, forming a conformationally entropic structure that enhances thermodynamic stability. These disulfide bridges are cross-links between molecules, reducing flexibility and stabilizing protein regions. The Disulfide by Design 2 v2.13 tool has been utilized to add disulfide bonds to the vaccine construct (Craig and Dombkowski, 2013). Pairs that were left behind with bond energy <2.2 kcal/mol, which is the standard range of energy of an unbroken disulfide bond, were selected.

#### 2.6.5 Docking of the vaccine constructs with immune receptors

To assess binding efficacy, the selected vaccine design was docked against six HLA alleles and Toll-like receptor 4 (TLR4) employing the ClusPro v2.0 server. ClusPro uses a docking method based on the Fast Fourier transform (FFT) to evaluate shape complementarity and desolvation energy, producing multiple docking conformations (Kozakov et al., 2017). The resultant docking was also verified by HawkDock v2 and PRODIGY. HawkDock combines molecular dynamics calculations with MM/GBSA free-energy calculations (Zhang et al., 2025), and PRODIGY calculates binding affinities using structural and thermodynamic properties (Xue et al., 2016). The visualization of the docked complexes was conducted using PyMOL v3.1 to detail conformational fitting (Yuan et al., 2017). In addition, the PDBSum software was utilized to generate 2D interaction diagrams and to highlight significant intermolecular contacts, such as hydrogen bonds, salt bridges, and hydrophobic interactions (Laskowski, 2001).

### 2.7 Normal mode-based structural analysis

The residual-level fluctuations and the flexibility of the docked complexes under physiological conditions (300 K, 1 atm) were measured using the internal coordinates normal mode analysis server (iMODS) (López-Blanco et al., 2014). This platform allows the basic dynamics to be determined by analyzing typical characteristic normal modes of motion in internal coordinates. Moreover, iMODS measures the molecule’s flexibility by determining B-factors, covariance matrices, deformability profiles, and eigenvalues. Deformability is a measure of how readily individual residues can undergo structural deformation. The normal mode eigenvalues are the stiffnesses of these motions, with the lowest eigenvalues being the softer collective motions that need less energy to occur.

### 2.8 Dynamic stability assessment of the vaccine-receptor complexes

To evaluate the conformational stability of the top-ranked vaccine–receptor complexes under explicit aqueous conditions, the Vc7-2FSE and Vc7-2Z65 complexes were selected for molecular dynamics simulations based on their superior docking performance, including favorable MM-GBSA binding energies, stronger binding affinities, lower dissociation constants, and stable intermolecular interaction profiles relative to the remaining docked complexes.

They were all simulated in Linux system using GROMACS v2024.2 (Abraham et al., 2015). The CHARMM36 all-atom force field has been used to calculate parameters required and to conduct the MD simulations (Vanommeslaeghe et al., 2012). The complexes have been placed in a cubic box filled with the TIP3P water model with a 1 nm buffer on each side. Counter-ions (Na^+^/Cl^−^) were introduced to neutralize the overall charge of the system and reproduce the physiological salt concentration. Energy minimization (50,000 steps) was then performed using the steepest-descent algorithm to remove steric clashes and unfavorable contacts. After energy minimization, the system has been equilibrated in 2 sequential stages. First, a constant number of particles (N), system volume (V), and temperature (T) (NVT) ensemble has been applied to stabilize the temperature, followed by a constant number of particles (N), system pressure (P), and temperature (T) (NPT) ensemble to maintain stable pressure and density. Each phase was carried out for 100 ps at 300K and 1 bar. The V-rescale thermostat and C-rescale barostat were used to regulate temperature and pressure, respectively. The PME (Particle Mesh Ewald) technique has been applied to account for long-range van der Waals and electrostatic interactions, using a 1 nm real-space cutoff. The simulation was performed with a time step of 2 fs for 150 ns. To assess the structural stability of complexes, trajectory analyses have been conducted, including radius of gyration (Rg), backbone root-mean-square deviation (RMSD), hydrogen bond assessment, root-mean-square fluctuation (RMSF), and solvent-accessible surface area (SASA).

### 2.9 Principal component analysis (PCA) and free energy landscape (FEL)

PCA has been conducted to verify the most common overall motions and conformational changes of complexes over 150 ns of MD simulation (David and Jacobs, 2014). PCA determines the main components by diagonalizing the covariance of Cα atom positional deviations after considering the alignment of all trajectories to a reference structure (Roy and Anbarasu, 2025). The resulting eigenvectors are the directions of collective motion, whereas the associated eigenvalues are the magnitudes. PCA was computed in the *gmx covar* module of GROMACS, and essential modes were analyzed in the *gmx anaeig* module to indicate the contribution of each principal component (PC) to the total motion and understand trajectories projection. The first PCs (PC1 and PC2), which captured the largest amount of motion, were analyzed to identify biologically important conformational changes.

FEL analysis has been performed to define the conformational space explored by the vaccine-target protein complexes during molecular dynamics simulations (MDS). FEL provides a thermodynamic description in which the probability of the observed conformation is correlated with the corresponding Gibbs free energy, making it easy to identify stable basins and transition states (Biswas and Anbarasu, 2025). The FEL is formed based on the basic subspace defined by PC1 and PC2, which represent the most noticeable aggregate movements of the complex. GROMACS was used to compute free-energy distributions as population density over the free-energy surface using the *gmx sham* script (Papaleo et al., 2009).

### 2.10 Dynamic cross-correlation analysis of residue motions

The cross-correlation map was used for examining the movements of the Cα atom in the vaccine-immune receptor complexes. The following equation was employed for calculating the correlation matrix for every complex:

$$Cij=\left(\Delta ,r,i,*,\;,\Delta ,r,j\right)/\left\{\sqrt{\left(\Delta ,r,i,*,\;,\Delta ,r,i\right)*\left(\Delta ,r,j,*,\;,\Delta ,r,j\right)}\right\}$$

Temporal covariance between atoms i and j is represented by the matrix Cij, where Δri and Δrj stand for the fluctuations in the i-th and j-th variables, respectively. Correlated motions were represented by positive values in the correlation matrix, whereas anti-correlated motions were indicated by negative values (Okamoto and Ando, 2023).

### 2.11 Codon optimization and virtual cloning

Vaccine development requires cloning and heterologous expression to produce as much recombinant protein as possible. The Java Codon Adaptation Tool (JCat) and EMBOSS Backtranseq were used to optimize codon sequences and invert codon sequences, respectively (Madeira et al., 2024). The GC content of 30%–70%, and the codon adaptation index (CAI) were both at a high level of 0.8 to ensure stability of the mRNA and effective translation (Grote et al., 2005). The SnapGene v8.2.1 program has been utilized to insert the maximized gene sequence into *E*. *coli* K-12 expression vector pET-28a (+). The recognition sites of the NheI and Bst API restriction enzymes at the 5′and 3′ends of the construct were included to allow accurate and effective cloning. The advantage of this design strategy is that it enables efficient insertion into the expression vector and high-level protein expression in bacterial systems (Alsaiari et al., 2024).

### 2.12 Immune simulation

A simulation of the immune system was conducted to determine the host’s immunological response to a foreign antigen. Following structural validation and stability assessment of the selected vaccine construct, immune simulation was performed to evaluate the predicted host immune response. The C-IMMSim web-based platform was used to analyze the final *Brucella* vaccine construct (Rapin et al., 2010). This server generates immunological profiles that characterize antigen-immune cell interactions. It determines immune responses utilizing computer methods and position-specific scoring matrices. The simulated vaccination activity used a three-dose regimen based on conventional vaccination schedules, which require at least 4 weeks between vaccination doses (Castiglione et al., 2012). The doses consisted of 1000 vaccine proteins and were administered at four-week intervals, with simulation times of 1, 84, and 168, where a time step of 1 represents a single injection (t = 0). The vaccination was given in three doses over 8 h, spread over more than 4 weeks. The default parameters were used to run the simulation, with 1100-time steps. The resulting immune profiles comprised antigen populations, interferons, antibodies, cytokines, and distinct sets of T cells, including helper, regulatory, and cytotoxic T cells.

## 3 Results

### 3.1 Pan-genome analysis and identification of potential vaccine candidates

**FIGURE 2:**
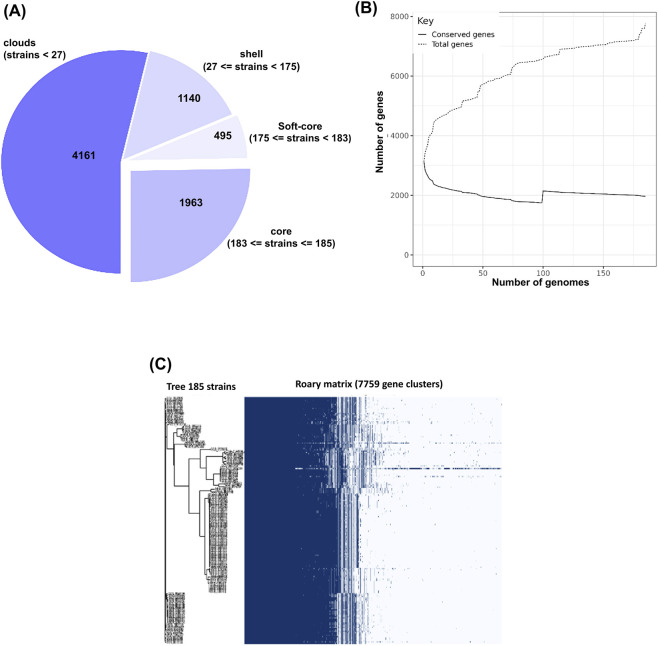
Genome-wide examination of core, accessory, and unique genes across *Brucella* spp.: **(A)** Pie chart depicting gene category distribution among strains. **(B)** Graph illustrating total vs. conserved genes. **(C)** Gene cluster matrix depicting the presence/absence of core and accessory genes.

Analysis of the whole gene repertoire across 185 annotated *Brucella* strains identified 1963 conserved core genes among 7759 gene clusters. As shown in Figure 2A, 495 were soft-core genes, 1140 were shell genes, and 4161 were cloud genes. The total number of genes vs. conserved genes across all genomes is shown in Figure 2B, and the gene presence-absence matrix is depicted in Figure 2C. Subcellular localization analysis using Gneg-Ploc, CELLO, and PSORTb identified several proteins associated with surface, membrane-associated, periplasmic, and intracellular localization. Candidate proteins were subsequently prioritized based on antigenicity, immunogenicity, solubility, conservation, and non-homology to the human proteome. Following screening with VaxiJen, SOLPro, and ANTIGENpro, five proteins met the predefined selection criteria and were selected for downstream analyses, as shown in Table 1. The subcellular localization of the selected proteins is summarized in Supplementary Table S1. Among the shortlisted proteins, BamA1 was consistently predicted to be an outer membrane-associated protein and was retained as a classical outer membrane vaccine target, whereas Tig, UreB, UreC1, and Der were predominantly predicted to be cytoplasmic/periplasmic proteins by multiple localization tools. However, candidate prioritization in the present study was not based solely on subcellular localization; additional criteria, including antigenicity, solubility, conservation, and immunological relevance, were also considered when selecting proteins. Notably, Tig has been reported to elicit protective immune responses, while urease-associated proteins have been extensively investigated as immunogenic vaccine antigens in bacterial pathogens (Vermoote et al., 2012; Cohen et al., 2019; Skakic et al., 2023). Although Der GTPase met the preliminary screening criteria, it was not retained in the final vaccine construct prioritization due to limited evidence of its relevance to vaccine-associated immunity (Hwang and Inouye, 2006).

**TABLE 1:** Antigenicity and solubility assessment of 12 proteins.

Protein name	NCBI accession number	AntigenPro	VaxiJen v2.0		SOLPro	
		Antigenic score	Antigenicity	Antigenic score	Solubility	Probability
Tig	CDL76300.1	0.747424	Probable Antigen	0.6427	Soluble	0.511052
BamA	GAA5662309.1	0.816554	Probable Antigen	0.6004	Soluble	0.611312
Omp10	WP_313829137.1	0.895552	Probable Antigen	0.7698	Insoluble	0.542727
BhuA	AIJ84416.1	0.93565	Probable Antigen	0.6175	Insoluble	0.582911
Omp16	P0A3S9.1	0.869937	Probable Antigen	0.5613	Insoluble	0.656245
UreB	AHN46974.1	0.932634	Probable Antigen	0.7863	Soluble	0.843894
YiaD	GAA5662259.1	0.92747	Probable Antigen	0.6876	Insoluble	0.816413
Der	WP_312472940.1	0.2785	Probable Antigen	0.5561	Soluble	0.618827
UreC1	SUW39063.1	0.279102	Probable Antigen	0.509	Soluble	0.609327
BamE	QMV26047.1	0.809495	Probable Antigen	0.4053	Insoluble	0.6943
SurA	GAA5662036.1	0.344424	Probable Antigen	0.5293	Insoluble	0.617594
OmpA	WP_250040970.1	0.946197	Probable Antigen	0.6928	Insoluble	0.87839

### 3.2 Immunogenic epitope profiling

#### 3.2.1 Assessment of Class-I MHC T Cell epitopes

Potential MHC class-I T cell epitopes from the five selected proteins were predicted using ProPred-I with proteasome filters and a 3% cut-off. A total of 18 epitopes in Tig, 19 epitopes in BamA, 5 epitopes in UreB, and 10 epitopes in UreC1 were obtained. High-affinity epitopes from IEDB were selected using a binding affinity criterion of 50 nM for IC50, which produced 64, 167, 20, and 53 epitopes in Tig, BamA, UreB, and UreC, respectively. Common epitopes were selected from both tools and evaluated for toxicity, immunogenicity, and antigenicity. The remaining epitopes were filtered and screened after 101 were identified as non-toxic. Out of these, 46 epitopes were selected for vaccine development due to their dual immunogenic and antigenic properties, as listed in Supplementary Table S2.

#### 3.2.2 Prediction of Class-II MHC T Cell epitopes

Class-II MHC T cell epitopes have been identified using the IEDB tool, and candidates with an IC50 <50 nM and a percentile score of <0.2 were selected. Consequently, 327 epitopes were identified across the five target proteins: 13 in Tig, 155 in BamA, 21 in UreB, and 75 in UreC1. After evaluation using the CD4^+^ T cell immunogenicity approach, 141 epitopes were identified, and a percentile rank <66 was used as the cutoff. Employing VaxiJen and ToxinPred to screen for toxicity and antigenicity, the list was further reduced to 51 non-toxic, antigenic epitopes (Supplementary Table S3) for vaccine development.

#### 3.2.3 Functional clustering analysis of HLA alleles

The chosen MHC class I and II epitopes have been assessed based on their IC_50_ values. A total of 46 class-I and 51 class-II MHC protein targets have been analyzed using MHCcluster. Each epitope was evaluated individually against all available HLA alleles. The interactions between the selected epitopes and their corresponding alleles were illustrated through a heat map (Supplementary Figure S1).

#### 3.2.4 Population coverage analysis of class-I and II MHC epitopes

Analysis of population coverage is significant in vaccine design owing to the polymorphism of HLA alleles. Epitope selection with demonstrated binding across individual HLA types is required, as HLA alleles vary in their ability to display T cell epitopes. Epitopes were chosen for their predicted high response rates and efficient presentation by different HLA molecules. The population coverage analysis indicated an average predicted coverage of approximately 96.27% across diverse geographic regions and ethnic groups, suggesting that the selected epitope repertoire exhibits broad HLA representation and may confer extensive global immunological coverage across diverse human populations (Table 2).

**TABLE 2:** Consolidated epitope prediction outcomes across four selected target proteins.

Population/Area	Coverage (%)		
	MHC-I	MHC-II	Combined
Central Africa	76.32	87.10	96.94
Central America	5.12	63.14	65.03
East Africa	77.60	90.58	97.89
East Asia	98.39	91.27	99.86
Europe	98.42	96.56	99.95
India	93.12	90.42	99.34
North Africa	88.05	94.64	99.36
North America	96.89	97.24	99.91
Northeast Asia	91.93	73.20	97.84
Oceania	96.16	73.48	98.98
South Africa	80.71	51.56	90.65
South America	88.90	75.54	97.29
South Asia	93.01	90.72	99.35
Southeast Asia	94.01	72.42	98.35
Southwest Asia	91.77	68.85	97.44
West Africa	84.40	94.51	99.14
West Indies	94.94	85.93	99.29

The regional variations observed in population coverage are primarily attributable to differences in HLA allele distributions and genetic diversity across distinct ethnic and geographic populations. Regions such as Europe, East Asia, and North America exhibited comparatively higher predicted coverage due to broader representation of the selected epitopes across prevalent HLA alleles, whereas lower coverage in certain regions may reflect reduced representation of specific HLA haplotypes within the selected epitope repertoire.

#### 3.2.5 Mapping of linear B Cell epitopes

B cell epitope prediction has been performed using BepiPred and ABCPred, thereby ensuring greater accuracy and confidence in the selected candidates. ABCPred analysis revealed 47 epitopes for Tig, 77 for BamA, 16 for UreB, and 60 for UreC1. Such initial predictions were subsequently cross-validated with BepiPred, which further predicted 15 epitopes in Tig, 29 in BamA, 6 in UreB, and 18 in UreC1. Upon comparing the results obtained with both tools, a total of 26 overlapping epitopes were identified and used as dependable consensus epitopes. These were widely anticipated epitopes considered in the design of the final vaccine molecule. B cell epitopes within the yellow region were regarded as potent B cell epitopes, as illustrated in Supplementary Figure S2.

#### 3.2.6 Epitope conservancy analysis

Epitope conservancy analysis was conducted to evaluate the cross-species conservation of the final shortlisted epitopes incorporated into the vaccine construct. The selected epitopes demonstrated high sequence conservation across representative *Brucella* spp., including *B. melitensis*, *B. abortus*, *B. suis*, *B. canis*, and *B. ovis*. The analysis revealed that most epitopes exhibited substantial conservation at an 80% sequence identity threshold, indicating that the identified immunogenic regions are evolutionarily conserved across diverse *Brucella* species. These findings further support the potential of the proposed construct as a broad-spectrum universal multi-epitope vaccine candidate against brucellosis.

### 3.3 Vaccine construct design

**FIGURE 3:**
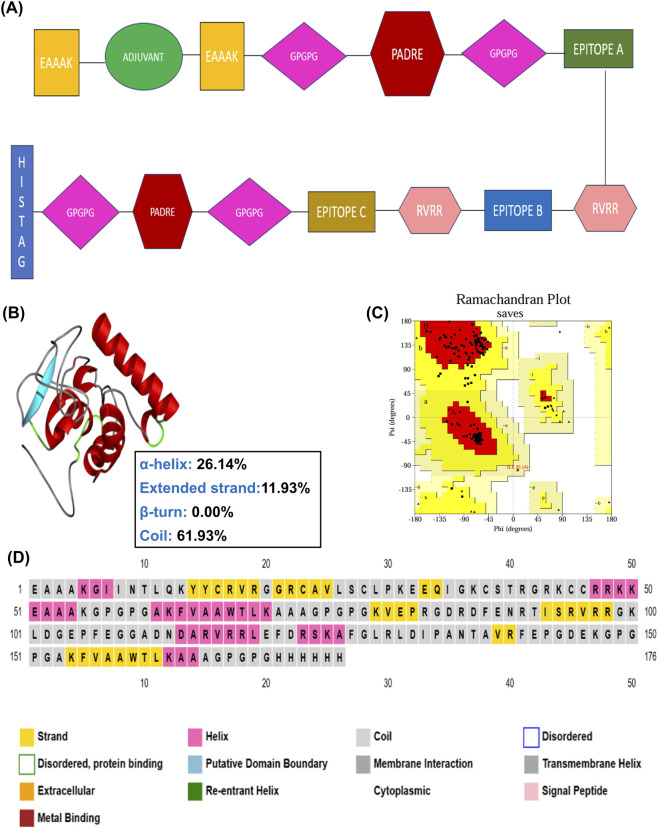
Final Vaccine Construct (Vc7). **(A)** Schematic representation of a multi-epitope vaccine construct. **(B)** 3D structure of Vc7 showing secondary elements. **(C)** Ramachandran plot showing 95.98% residues in favored regions. **(D)** Visualization of the Vc7 sequence components.

The final vaccine construct was designed to include all distinct and overlapping epitopes identified by the class-I/II MHC and B cell prediction analyses, as consolidated in Table 3. Several adjuvants, such as L7/L-12 ribosomal proteins, HBHA, β-defensin, and conserved HBHA, were used to enhance the vaccine’s immunogenicity. The adjuvants were incorporated into the vaccine’s ordered epitope arrangement. In addition to the adjuvants, the epitopes were connected by linkers such as RVRR, and PADRE sequences were connected by linkers such as GPGPG. The adjuvants were linked to both ends of the construct via EAAAK, thereby enhancing the vaccine’s immunogenicity, stability, and potency. By enhancing T helper cell activation, PADRE improves vaccine efficacy. A C-terminal His tag was also added to facilitate purification and enhance the construct’s overall efficacy. Figure 3A depicts the vaccine design.

**TABLE 3:** Consolidated epitope prediction outcomes across four selected target proteins.

Protein name	MHC-I T cell		MHC-II T cell		B cell	Position
	Epitope sequence	Position	Epitope sequence	Position	Epitope sequence	
Tig	SSTRTFETK	156-164	AREEYRKLAERRVRL	347-361	ETLNEGLKREIKVVVP	7-22
	EAAREEYRK	345-353	DGKADFVFSLNYEVL	108-122	VLPAIEVKDFSKIAVT	121-136
	KLAERLETA	29-37	GKADFVFSLNYEVLP	109-123	GKLDGEPFEGGADNDA	180-195
	EVLPAIEVK	120-128	KADFVFSLNYEVLPA	110-124	LGSGQFIPGFEEQLIG	199-214
	FIPGFEEQL	204-212	ADFVFSLNYEVLPAI	111-125	PAEYGAAHLAGKEATF	228-243
	KVITVTFPA	221-229	LDGKADFVFSLNYEV	107-121	GVEVTEEELQRAVYDQ	373-388
	RKLAERRVR	352-360	DEQVKRIASSTRTFE	148-162		
	ITVTFPAEY	223-231	FVFSLNYEVLPAIEV	113-127		
	LVDAEFNNI	314-322				
	AENEDRVTI	168-176				
	ETARGRARI	35-43				
	YEVLPAIEV	119-127				
	DEKVITVTF	219-227				
BamA1	FVIRREFDV	376-384	FGPLRFDYAFPIAKA	755-769	ARTVDLGQGRVNVVYE	165-180
	NEYTITITV	264-272	PFGPLRFDYAFPIAK	754-768	GYADFRVISSNAVLDP	246-261
	KTRDFVIRR	372-380	GPLRFDYAFPIAKAD	756-770	KVEPRGDRDFENRTIS	334-349
	SLGIRGAVF	708-716	SPFGPLRFDYAFPIA	753-767	YIQRIEIRGNDKTRDF	361-376
	MEADVEAIK	142-150	PLRFDYAFPIAKADT	757-771	EFDVNEGDAFNQVMVQ	381-396
	FDYAFPIAK	760-768	ASPFGPLRFDYAFPI	752-766	TVNISTAPGSEPDQVI	410-425
	STNEYTITI	262-270	LGYRLSAGFDVFRRT	495-509	DVVEKSTGEFSIGGGY	429-444
	ESLGIRGAV	707-715	LRFDYAFPIAKADTD	758-772	GGYTTGGESPGAQVEA	442-457
	TSADIDAAV	70-78	GYADFRVISSNAVLD	246-260	AITERNFLGRGQYIRI	458-473
	DEETLRRFY	234-242	YFLGYRLSAGFDVFR	493-507	YIRISAGAGQDDMRNY	470-475
	RRLEALDFF	400-408	YADFRVISSNAVLDP	247-261	YGLSFTEPYFLGYRLS	485-499
	FSAGIAYNL	535-543	RGYADFRVISSNAVL	245-259	KFTQEFAGLGGDAKYI	599-614
	GPLRFDYAF	756-764	PYFLGYRLSAGFDVF	492-506	TFKGNYYKTLSQEADI	617- 632
	TYINGTAEV	690-698	NRGYADFRVISSNAV	244-258	EVQFPMPLVPESLGIR	697-712
	ATMEADVEA	140-148	ADFRVISSNAVLDPS	248-262		
	LRFDYAFPI	758-766	DFRVISSNAVLDPST	249-262		
	KVQNFNFGV	773-781	EPYFLGYRLSAGFDV	491-505		
	FTSADIDAA	69-77	FRVISSNAVLDPSTN	250-264		
	RLSAGFDVF	498-506	ADIDAAVKRLFAMGL	72-86		
	AKEIEDSVL	311-319	DIDAAVKRLFAMGLF	73-87		
	EPYFLGYRL	491-499	LRSSISYSLTYNSID	573-587		
	GQGRVNVVY	171-179	VDFVGNQAFSSRRLR	192-206		
	EFGDDGVRI	645-653	LGRGQYIRISAGAGQ	465-479		
			LFSDVRINQSGSTLV	86-100		
			MSVALVASGTAALSL	16-30		
			FLGRGQYIRISAGAG	464-478		
			TDKVQNFNFGVSTKF	771-785		
			FSDVRINQSGSTLVV	87-101		
			SDVRINQSGSTLVVN	88-102		
			GRGQYIRISAGAGQD	466-480		
			DVRINQSGSTLVVNV	89-103		
			RGQYIRISAGAGQDD	467-481		
			GQYIRISAGAGQDDM	468-482		
			DMRNYGLSFTEPYFL	481-495		
			VRINQSGSTLVVNVT	90-104		
			IANVDFVGNQAFSSR	189-203		
			SALAMSVALVASGTA	12-26		
UreB	RFIFGFNNL	111-119	LEFDRSKAFGLRLDI	72-86	YVLAKDPIEINQGRPR	29-44
	EIAAERAEK	137-145	AGKRFIFGFNNLVDG	108-122	DIPANTAVRFEPGDEK	85-100
	REIAAERAE	136-144			EKLGFKSCKSGGKDAK	144-159
	YQPNREIAA	132-140				
	FIFGFNNLV	112-120				
UreC1	PAIPEDIAF	326-334	GKHSMILNNAMPQME	524-538	HVHFISPQQVDEALNA	136-151
	LWNPAFFGV	436-444	KHSMILNNAMPQMEV	525-539	DLVLWNPAFFGVKPDM	433-448
	YEVRADGEL	544-552	GIGKHSMILNNAMPQ	522-536	SQAAMDEGLREKIGVD	495-510
	TEGAGGGHA	275-283	HSMILNNAMPQMEVD	526-540		
	QAMGRVGEM	364-372				

The secondary structure is shown in Figure 3B, and the Ramachandran plot is shown in Figure 3C. The composition of the secondary structure is represented in Figure 3D. The sequences of the 12 different vaccine constructs are provided in Supplementary Table S4.

### 3.4 Evaluation of the structural and chemical attributes of the vaccine designs

All 12 constructs were evaluated for antigenicity, solubility, allergenicity, and other physicochemical properties, as shown in Table 4. Except for Vc12, all the constructs were non-allergenic, as predicted by AlgPred and AllerTOP. All constructs are antigenic and soluble, as determined by antigenicity tests with VaxiJen and AntigenPRO and solubility tests with SOLPro. The theoretical isoelectric values (pI) varied from 5.59 to 10.03, and the molecular weight ranged from 18 to 40 kDa. The instability index criterion should be greater than 40; since Vc2 and Vc10 are <40, they are excluded as potentially unstable. The nine constructs with high aliphatic indices (ranging from 50 to 80) exhibited higher thermostability. A favorable hydrophilic profile conducive to water interactions and solubility was indicated by negative GRAVY scores. These assessments led to the selection of Vc (1, 3-9, and 11) constructs for further structural modeling and docking studies.

**TABLE 4:** Physicochemical profiling of 12 designed vaccine constructs.

Vaccine	Probable non-allergen		Probable antigen		Solubility probability	Molecular weight	Theoretical pI	Instability index	Aliphatic index	GRAVY
Server used	AlgPred	AllerTOP v2.1	VaxiJen v2.0	ANTIGENPro	SolPro	ProtParam	ProtParam	ProtParam	ProtParam	ProtParam
V_C_1	Non-Allergen	Probable Non-Allergen	0.8065	0.580996	0.797972	31535.2	5.92	34.68	74.36	−0.616
V_C_2	Non-Allergen	Probable Non-Allergen	0.8172	0.552766	0.856228	30279.83	5.59	40.29	78.01	−0.575
V_C_3	Non-Allergen	Probable Non-Allergen	0.9875	0.454321	0.997452	18930.6	10.03	29.03	57.84	−0.731
V_C_4	Non-Allergen	Probable Non-Allergen	0.7406	0.723468	0.555898	27209.89	5.96	21.91	79.12	−0.283
V_C_5	Non-Allergen	Probable Non-Allergen	0.7937	0.570663	0.800045	31398.05	5.84	33.76	74.62	−0.607
V_C_6	Non-Allergen	Probable Non-Allergen	0.8028	0.554432	0.855631	30279.83	5.59	39.25	78.01	−0.575
V_C_7	Non-Allergen	Probable Non-Allergen	0.9644	0.451213	0.997134	18930.6	10.03	27.36	57.84	−0.731
V_C_8	Non-Allergen	Probable Non-Allergen	0.7251	0.717256	0.552863	27209.89	5.96	20.79	79.12	−0.283
V_C_9	Non-Allergen	Probable Non-Allergen	0.81	0.5	0.790908	31398.05	5.84	35.46	74.62	−0.607
V_C_10	Non-Allergen	Probable Non-Allergen	0.8196	0.482892	0.84928	30279.83	5.59	41.01	78.01	−0.575
V_C_11	Non-Allergen	Probable Non-Allergen	0.9914	0.621259	0.997457	18930.6	10.03	30.18	57.84	−0.731
V_C_12	Non-Allergen	Probable Allergen	0.7432	0.73245	0.549772	27209.89	5.96	22.68	79.12	−0.283

### 3.5 Computational modeling and structural validation

The 3D models were created using an *ab initio* approach with trRosetta, since no template for the vaccine was available. The models were then refined through structure optimization and validated. Based on these quality determinations, the vaccine candidate of greatest promise was Vc7. The structure validation showed that Vc7 was the best vaccine construct, with a quality factor of 91.9 and a Ramachandran-favored region of 95.9%. GalaxyRefine has been used to refine the models. The quality parameters of vaccine constructs are summarized in Supplementary Table S5. The final vaccine constructs were found to have comparable backbone flexibility with an average S^2^-value of 0.75. Only the Vc7 and Vc11 structures passed, according to Verify 3D, whereas Vc1, Vc3, Vc4, Vc5, Vc6, Vc8, and Vc9 shows structural violations. Figure 3B presents the secondary-structure analysis of Vc7 using SOPMA. Although both Vc7 and Vc11 met the structural validation criteria, Vc7 exhibited comparatively superior physicochemical stability, lower backbone flexibility, improved structural quality metrics, and stronger receptor-binding interactions, thereby leading to its selection for downstream docking, molecular dynamics simulation, immune simulation, and cloning analyses.

### 3.6 Structural mapping of conformational B Cell epitopes

The final vaccine construct has been found by the ElliPro platform to harbor five discontinuous (conformational) B cell epitopes. These epitopes had moderate to high levels of surface accessibility and antigenic capacity, with 4–43 amino acid lengths and prediction scores of 0.506–0.709, as indicated in Supplementary Table S6. A visual representation of the epitopes predicted, with particular attention to their spatial distribution within the vaccine model, is provided in Supplementary Figure S3.

### 3.7 Disulfide engineering

Disulfide bonds were introduced to enhance the vaccine’s conformational stability by covalently linking specific residues. The 3D structure of the vaccine has been studied, along with 21 pairs of residues that could form disulfide bridges as provided in Supplementary Table S7. A residue pair, Ala-Gly, was chosen to mutate into cysteines. This would provide highly advantageous, constitutively stable disulfide bonds (Supplementary Figure S4), thereby enhancing the vaccine’s integrity under physiological conditions.

### 3.8 Computational docking analysis

**FIGURE 4:**
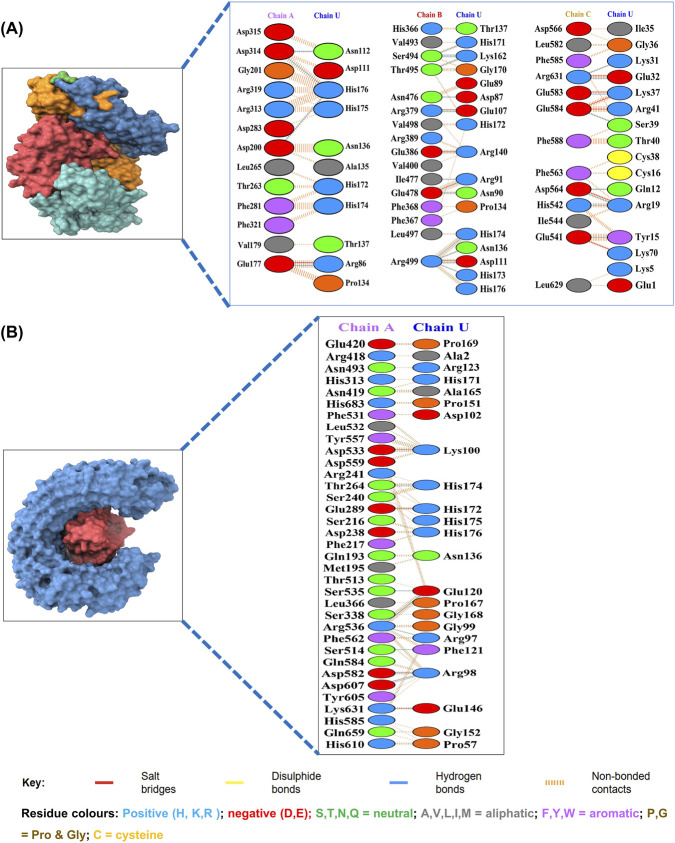
Vc7 docked with target proteins (red color denotes the vaccine in the 3D structure). **(A)** 3D Docked complex of Vc7-2FSE and intermolecular interactions. **(B)** 3D Docked complex of Vc7-2Z65 and intermolecular interactions.

Vc7 was docked with several immunological targets, including TLR4 and HLA alleles, to determine their binding affinities. Table 5 shows that the highest binding energy with 2FSE was −135.24 kcal/mol. The binding affinity ΔG (kcal/mol) and the dissociation constant (K_d_) were also used as supplements to these findings. When 2FSE and 2Z65 were docked to Vc7 (Table 6), several interactions were observed. The four chains that comprise 2FSE are chains A, B, C, and E. Chain U (vaccine Vc7) and Chain A are bound primarily by salt bridges and hydrogen bonds, with one salt bridge and 102 non-bonded contacts. The vaccine complex did not interact with chain E. Figure 4 shows the interactions between Vc7 and target proteins. These reactions suggest that Vc7 binds well to 2FSE, which could help boost immune responses and therapeutic outcomes. Chain B and chain U interaction showed 12 hydrogen bonds, four salt bridges, and 110 unbonded contacts. Chain C is connected to chain U using six salt bridges, 103 non-bonded contacts, and seven hydrogen bonds. The second binding was the most important, with 2Z65 and a binding energy of −96.18 kcal/mol, a binding affinity of −18 kcal/mol, and 2.40E-13 K_d_. Several interactions were observed when 2Z65 was docked with Vc7. The overall count of intermolecular H-bonds, salt bridges, and non-bonded contacts observed between Vc7 and 2Z65 was six, seven, and 193, respectively. Figure 4A,B demonstrate the interactions among 2FSE, 2Z65, and Vc7. Residue-level interaction analysis further demonstrated that the majority of receptor interactions involved predicted epitope residues, whereas linker, PADRE, adjuvant, and His-tag residues contributed comparatively fewer stabilizing contacts. In the 2FSE-Vc7 and 2Z65-Vc7 complexes, epitope-associated residues predominantly participated in hydrogen bonding, salt bridges, and non-bonded interactions, indicating that the designed immunogenic regions actively contributed to receptor recognition and binding stability. Additional interactions involving the linker and adjuvant regions may further stabilize the docked complexes. Detailed residue-level interaction information and the corresponding vaccine components are provided in Supplementary Table S8. The docking results showed that Vc7 strongly interacts with 2FSE and 2Z65, which could improve Vc7’s immune response and therapeutic potential.

**TABLE 5:** Docking scores and binding energies of Vc7 against immune receptors.

Vaccine construct	Target ID (PDB)	Binding energy (MM-GBSA method) (kcal/mol)	Binding affinity ΔG (kcal/mol)	Dissociation constant (K_d_)	Docking score
Vc7	1A6A	−95.72	−8.9	3.00E-07	−957.4
	1H15	−135.08	−14.4	2.70E-11	−934.7
	2FSE	−135.24	−17.1	6.50E-14	−1083.1
	2Q6W	−104.73	−15.4	5.10E-12	−954.4
	2SEB	−83.15	−8.8	3.70E-07	−937.3
	2Z65	−96.18	−18	2.40E-13	−983.5
	3C5J	−85.8	−13.6	1.10E-10	−961.9

**TABLE 6:** Interaction statistics of the docked vaccine-immune receptors.

Protein	Chains	No. of interface residues	Interface area (A^2^)	No. of salt bridges	No. of hydrogen bonds	No. of non-bonded contacts
2FSE	A-U	13:11	579:654	1	5	102
	B-U	16:17	866:813	4	12	110
	C-U	13:16	761:761	6	7	123
2Z65	A-U	35:23	579:654	6	7	193

### 3.9 Normal mode analysis

Normal Mode was estimated on the iMODS server, including elastic network characteristics, eigenvalues, variance and covariance analyses, B-factors of atoms in the complex, and their flexibility and dynamic features. The B-factor values in the first plot (Supplementary Figure S5A) indicated moderate B-factors for most residues, with localized peaks, compared to the Vc7 2Z65 complex (Supplementary Figure S5A), which showed more pronounced fluctuations and larger regions of flexibility, with a strong increase in B-factor towards the C-terminal region. Likewise, the deformability plot revealed RMS oscillations in the complex with Vc7-2FSE, indicating high flexibility that could enable efficient binding (Supplementary Figure S5B). As shown in Supplementary Figure S5C, the Vc7-2FSE complex had a slightly larger eigenvalue of 7.386397e-05 than the Vc7-2Z65 complex, whose eigenvalue was 3.703221e-05. This indicates that the Vc7-2FSE complex was relatively more stable yet adaptable, with increased resistance to deformation and structural rigidity. The variance analysis revealed the contribution of the mode of individual values and the cumulative mode contribution (Supplementary Figure S5D), indicating a balance between flexibility and structural integrity. The increased contribution of the first few low modes leads to reduced flexibility and more stable collective behavior. The covariance matrix of Vc7-2Z65 complex (Supplementary Figure S5E) has poor correlation between domains with enhanced anti-correlated motions, which is associated with poor stability, but the covariance matrix of Vc7-2FSE (Supplementary Figure S5E) has strong, well-organized blocks of positive correlation with limited anti-correlated regions, indicating good stability with coordinated residue movements. In both complexes, the covariance (Supplementary Figure S5F) was correlated, uncorrelated, and anti-correlated. Lower-density areas were observed, suggesting greater stability. Due to its versatility and consistent binding to 2FSE and 2Z65, this evidence suggests that Vc7 is a vaccine candidate.

### 3.10 MD simulation

The stability of vaccine-receptor molecules has been examined using a 150 ns MD simulation. Structural stability was evaluated by tracking variations in RMSD, RMSF, Rg, SASA, and intermolecular H-bonds throughout the trajectory. The combination of these parameters gives information concerning the structural stability, conformational flexibility, molecular compactness, solvent exposure, and intermolecular interactions of the complexes.

#### 3.10.1 Root mean square deviation (RMSD)

**FIGURE 5:**
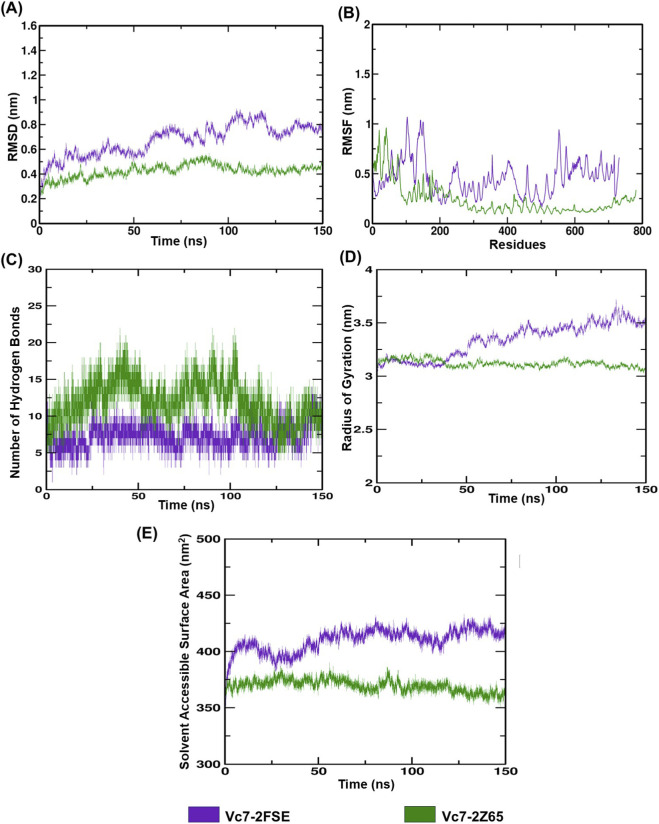
Molecular dynamics simulation analysis of Vc7-2FSE and Vc7-2Z65. **(A)** Root mean square deviation (RMSD). **(B)** Root mean square fluctuation (RMSF) associated with the number of residues. **(C)** Intermolecular H-bond. **(D)** Radius of gyration. **(E)** Solvent accessible surface area (SASA).

To compute the stability and dynamics of simulated complexes, the backbone RMSD was calculated over the 150 ns trajectory. This is the constant RMSD profile, suggesting that the complexes were structurally intact under dynamic conditions. The Vc7-2FSE and Vc7-2Z65 complexes exhibited stable dynamics, with mean RMSD values of 0.67 nm and 0.42 nm (Figure 5A). This structural stability is critical for maintaining the functional integrity of the vaccine-receptor complexes and may contribute to their effective immunogenic recognition and immune activation potential.

#### 3.10.2 Root mean square fluctuation (RMSF)

To evaluate residue-level conformational flexibility in the vaccine-receptor complexes, the RMSF values of the Cα atoms were computed. RMSF quantifies the mean positional variations of individual Cα atoms with respect to their average position during the simulation, and therefore characterizes the natural flexibility of particular regions of the complex. Low RMSF values indicate less local variability and greater structural stability. The Vc7-2Z65 complex showed smaller RMSF variations, with most occurring in loop regions and during the early equilibration phase. Other residues showed small changes, suggesting the general stability of the Vc7-2Z65 complex (Figure 5B). Conversely, the Vc7-2FSE complex had relatively larger RMSF values, indicating that it is more flexible than Vc7-2Z65.

#### 3.10.3 Hydrogen bonds

The H-bonding associations within the vaccine-receptor complexes were examined using MD simulations, which play a role in providing intramolecular stability and intermolecular recognition (Figure 5C). The simulations showed that the Vc7-2FSE complex had 7.3 average of intermolecular hydrogen bonds, and the Vc7-2Z65 complex had 11.9 average of intermolecular H-bonds. Large hydrogen-bond networks in these two complexes helped provide structural stability and reduce their size. The reported interactions promote the effective display of the multi-epitope vaccine by MHC molecules, thereby promoting effective T cell activation. Furthermore, the strong interactions between the vaccine and TLRs suggest the ability to trigger innate immune signaling pathways, including cytokine and chemokine secretion. These profiles of protein-protein interactions strongly indicate that the designed vaccine would effectively interact with key immune receptors, stimulate coordinated innate and adaptive immune responses, and serve as a promising vaccine candidate.

#### 3.10.4 Radius of gyration (Rg)

The Rg analysis quantifies the spatial distribution of atomic coordinates over the course of the simulation and provides a measure of structural compactness for vaccine-immune receptor complexes. Figure 5D also indicates that the Vc7-2FSE and Vc7-2Z65 complexes displayed stable conformational behavior with mean Rg values of 3.33 and 3.11 nm, respectively. Rg trajectories of both complexes were similar, with slight fluctuations, indicating no significant conformational expansion or collapse during the simulation. The fact that the Rg values are stabilized also indicates that the vaccine constructs adopt a well-structured, compact structure when they bind to the corresponding immune receptors. Generally, the Rg analysis indicated that all vaccine-immune receptor complexes retained their structural integrity and compactness, demonstrating receptor engagement and minimal conformational drift.

#### 3.10.5 Solvent accessible surface area (SASA)

SASA was conducted to assess solvent-accessible surfaces of vaccine-immune receptor complexes after 150 ns of MD simulations. Both systems had equal SASA values, with means of 410 nm for the Vc7-2FSE complex and 369.58 nm for the Vc7-2Z65 complex. The small change between the simulations suggests the presence of steady dynamic behavior (Figure 5E). The constant SASA values indicate that all the complexes have similar solvent accessibility, which is critical for sustaining protein-protein interactions and further signal transduction.

### 3.11 PCA and FEL analysis

**FIGURE 6:**
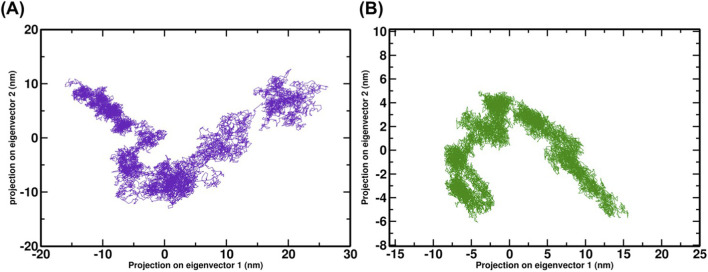
Principal component analysis of protein motion phase space during a 150 ns simulation. **(A)** Vc7-2FSE. **(B)** Vc7-2Z65.

To describe the major large-scale motions and dynamic properties of the vaccine-immune receptor complexes of Vc7-2FSE and Vc7-2Z65, the PCA was conducted (Figures 6A,B). The most important directions of motion are PC1 and PC2, which explain the significant conformational differences among the complexes. The clustering of trajectories in the low-energy regions may indicate that both the vaccine and the receptor systems reached a state of structural stability and dynamic equilibrium.

**FIGURE 7:**
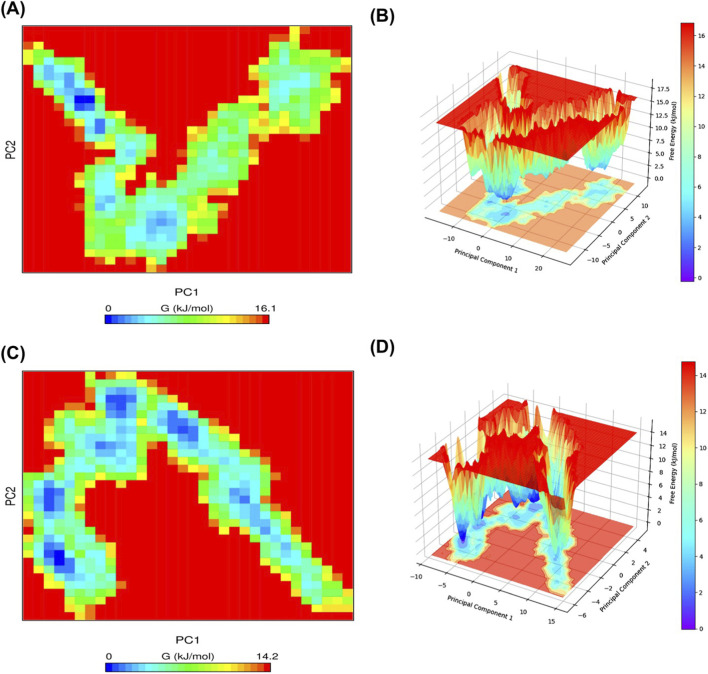
Gibbs free energy landscapes. **(A,B)** 2D and 3D Gibbs free energy landscapes of Vc7-2FSE. **(C,D)** 2D and 3D Gibbs free energy landscapes of Vc7-2Z65.

The corresponding FEL maps (Figures 7A–D) displayed well-defined low-energy basins that represent the most thermodynamically favorable conformational states sampled during the simulations. These basins demonstrate that the vaccine-immune receptor complexes achieved stable energy minima, reflecting energetically favorable, compact conformations. The color gradient, ranging from red (higher energy, less stable) to blue (lower energy, more stable), further illustrates these stability differences.

### 3.12 DCCM analysis

The concordant and discordant motions between residues and domains were quantified using DCCM analysis, providing insight into how these domains and residues are dynamically coupled and how they vary as a group. Positive correlation values indicate synchronized or parallel movement, whereas negative correlation values indicate opposed or antiparallel movement. The resulting DCCM plots are realistic representations of the dynamic correlations within the vaccine-immune receptor complex, with their intensity and direction indicated by a color gradient. The red areas in Vc7-2FSE (Supplementary Figure S6A) and Vc7-2Z65 (Supplementary Figure S6B) complexes are positively correlated motions, whereas the blue areas are the anti-correlated motions.

### 3.13 Optimization of codon usage and computer-based gene cloning

**FIGURE 8:**
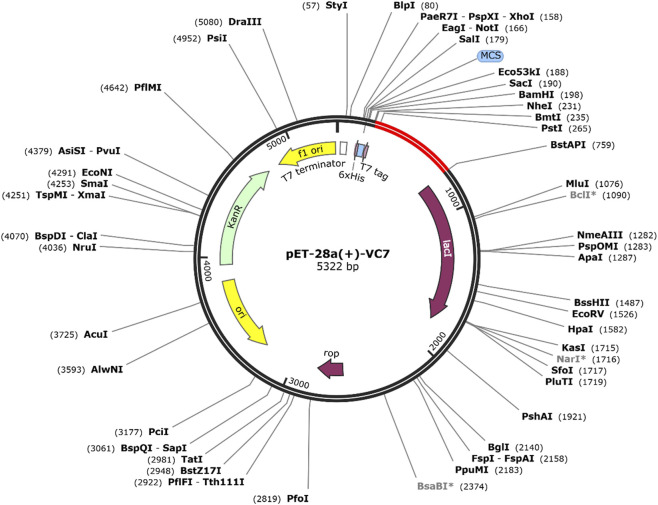
*In silico* cloning of the Vc7 construct into the pET-28a (+) expression vector, the inserted gene segment is indicated in red.

The JCat platform was used to optimize the Vc7 for heterologous expression in the *E. coli* K-12. EMBOSS Backtranseq was used to reverse-transcribe the protein sequence into a DNA sequence that complied with the codon usage preferences of the bacterial host. The optimized sequence, with a CAI of 1 and a GC content of 55.30%, exhibited favorable characteristics for *E. coli* expression. 528 base pairs were measured in the final optimized construct. Using the restriction enzyme sites NheI and BstAPI for directed cloning, the codon-optimized gene was integrated into the pET-28a (+) expression vector (Figure 8). SnapGene software was used to replicate the cloning process, ensuring correct gene integration. Construct’s suitability for experimental *E. coli* expression is confirmed by this *in silico* procedure.

### 3.14 Immune simulation

**FIGURE 9:**
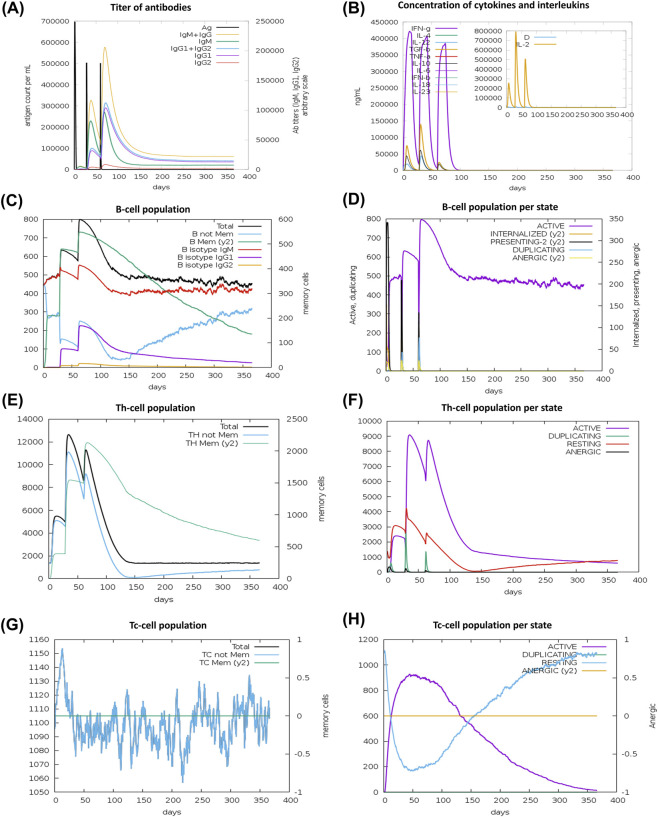
Immunological response maps for the human body following *Brucella* vaccine injection **(A)** Multicolored immunoglobin concentration upon antigen exposure (vertical black line). **(B)** Cytokine production. **(C)** B lymphocyte population. **(D)** B lymphocyte population per state. **(E)** Helper T cell concentration. **(F)** Helper T cell concentration per state. **(G)** T cytotoxic cell population. **(H)** T cytotoxic cell population per state.

C-IMMSim tool was employed to predict immunological responses following vaccination. The results showed that antibody-mediated and cellular immune responses were triggered by the vaccine. IgM, IgG1, and IgG2 concentrations were enhanced after three doses, resulting in a significant rise in antibody titers (Figure 9A). Following each dose, cytokine levels, specifically IFN-γ and IL-2, increased, although antigen levels fell (Figure 9B). B cell populations, such as memory B cells and IgM-producing B cells, increased after the second and third doses (Figures 9C,D). T helper cells and T lymphocytes showed increased activity and memory cell formation, particularly after the second and third doses (Figures 9E,G). In both active and resting phases, T cell population per state varied significantly (Figures 9F,H).

## 4 Discussion

Brucellosis is a serious global health concern and contributes to numerous infections, particularly for immunocompromised patients (Pellegrini et al., 2022; Dadar et al., 2025). Despite its importance, there is currently no vaccine available that effectively prevents *Brucella* infections (Tian et al., 2024). A potent vaccine should ideally elicit humoral and cell-mediated immune responses against the pathogen (Amanna and Slifka, 2011). *In silico* approaches offer a faster and more reliable way of developing vaccines than traditional approaches (María et al., 2017; Sunita et al., 2020). Therefore, our research was strategically designed for developing a multi-epitope vaccine candidate against *Brucella* spp. utilizing a comprehensive computational method.

In this study, the integration of core genome analysis with the reverse vaccinology approach overcomes the limitations associated with species-specific antigen selection and reliance on predefined protein subsets of *Brucella* spp. as reported in previous works by Zai et al. (2021); Yin et al. (2023); Nora and Khadidja (2024); Shi et al. (2024). The present approach enhances the potential for cross-species immune coverage and reduces the risk of antigenic variability by ensuring 100% conservation of the core proteome prior to epitope mapping. Moreover, in contrast to the previous pan-genome analysis reported by Hisham and Ashhab (2018), which was confined to antigen identification and subcellular localization profiling of *Brucella* spp., the present investigation extends to comprehensive multi-epitope vaccine construction, evaluation of physicochemical characteristics, structural validation, and assessment of stability dynamics of vaccine-immune receptor complexes. This integrated strategy enhances the robustness of candidate prioritization and strengthens the translational potential of the proposed vaccine candidate against *Brucella* spp.

Immunoinformatics approaches integrated with pan-genome analysis can be considered a crucial component in vaccine design to develop effective multi-epitope vaccines against pathogens (Dong et al., 2020; Jalal et al., 2023; Alhassan, 2024). Pan-genome analysis plays a key role in ensuring universal homogeneity for broad-spectrum vaccine candidates against *Brucella* spp. (Rida et al., 2022). In our study, 185 complete annotated genomes were selected from a total of 1729 *Brucella* genomes from the NCBI database. Core proteins were evaluated based on their antigenicity, subcellular localization, solubility, and non-homology to the human proteome (Priyamvada et al., 2024). Only four proteins were shortlisted as promising immunization targets: Tig, BamA, UreB, and UreC1.

These shortlisted proteins are functionally associated with essential mechanisms for bacterial survival and host adaptation. Tig acts as a ribosome-associated molecular chaperone involved in nascent protein folding and stress adaptation under hostile intracellular conditions (Deeng et al., 2016). BamA is a central component of the β-barrel assembly machinery responsible for outer membrane protein biogenesis and for maintaining membrane integrity in Gram-negative bacteria, thereby contributing to bacterial viability and host interactions (Lundquist et al., 2021). UreB and UreC1 are catalytic components of the urease enzyme complex associated with acid resistance, nitrogen metabolism, and intracellular persistence within the host environment (Sangari et al., 2007; Carter et al., 2011). The functional importance and conservation of these proteins further support their suitability as potential vaccine targets against *Brucella* spp.

The vaccine’s immunogenicity could be improved by incorporating B and T cell epitopes (Chen et al., 2021). Common epitopes from core proteins, which are significant for *Brucella* pathogenicity, were chosen according to their immunogenic, antigenic, and non-toxic properties (Maharaj et al., 2021). A total of 26 B cell epitopes and 97 T cell epitopes, comprising 46 MHC class-I and 51 MHC class-II epitopes, were identified, indicating broad immunogenic coverage and strong potential for eliciting both humoral and cellular immune responses. Furthermore, epitope conservancy analysis demonstrated that the final shortlisted epitopes incorporated into the vaccine construct were highly conserved across representative *Brucella* spp. Conserved epitopes are less susceptible to antigenic variation and may facilitate cross-protective immune responses against genetically diverse strains. The observed high conservation of the selected epitopes, therefore, strengthens the rationale for developing a universal multi-epitope vaccine candidate capable of providing broad-spectrum protection against brucellosis. A greater number of epitopes combined with adjuvants, PADRE sequences, 6-histidine (His) tags, EAAAK, GPGPG, and RVRR linkers were employed in the final vaccine design to enhance efficacy (Onile et al., 2020). Adjuvants attached to the N-terminal region of the vaccine were connected employing EAAAK. The GPGPG linker, which contains glycine, enhances the vaccine’s solubility and flexibility (Roy et al., 2024). It was used to link the epitopes with the PADRE. It was also included to determine the increase in the activity of helper T cell (Priyamvada and Ramaiah, 2023). Also, the furin-sensitive RVRR was used for connecting epitopes.

Adjuvants must be included to enhance the immune-stimulating capability of vaccines. Pathogen-derived adjuvants such as β-defensin, heparin-binding hemagglutinin (HBHA), HBHA conserved, and ribosomal protein L7/L12 were used in the study because they have a known capacity of regulating immune responses (Maurya et al., 2023). T cell is activated by Adjuvant HBHA, and INF-γ is produced (Pethe et al., 2001). β-defensin is an antimicrobial peptide that also contributes a lot to innate immunity (He et al., 2006). The L7/L12 ribosomal protein also plays an essential role in regulating the translation process and is required to mediate the GTPase activity of the elongation factor (Carlson et al., 2017). These molecular adjuvants facilitate T cell priming, exert antimicrobial effects, and help regulate protein production, thereby enhancing the host immune defense (Jalal et al., 2021). Compared with conventional adjuvants, which can cause unwanted side effects, protein-based adjuvants induce a specific immune response, leading to a targeted, more effective response (Lavelle and McEntee, 2024). Moreover, the His tag incorporated into the vaccine construct facilitates protein purification and protein stability, as well as increasing antigen interaction with antigen-presenting cells (Sun et al., 2011).

Twelve vaccine constructs were developed and analyzed in terms of physicochemical properties (GRAVY, solubility, instability index, allergenicity, and antigenicity) (Alnajran et al., 2025). Out of 12 vaccine constructs, 9 were found to have stability, high thermal stability, and instability indices of less than 40. The GRAVY scores are negative, indicating that the proteins are hydrophilic and very soluble (Gul et al., 2023). Hydrophilic residues interact with immune cells, and hydrophobic residues govern protein folding and antibody binding. The adjuvant L7/L12 ribosomal protein was included in all the chosen vaccine constructs and helped to stabilize the structure along with linkers, PADRE, and His Tag. The Tr-Rosetta server system has been utilized to model the finalized vaccine constructs. Vc7 was the most promising *Brucella* vaccine candidate based on its structural stability and backbone dynamics (Ismail et al., 2022).

Vc7 has 176 amino acids, and the molecular weight is 18,930.6 Da. Proteins with molecular weights below 110,000 Da are preferred for vaccine development due to their efficient expression and purification relative to larger proteins. Physicochemical studies demonstrated that Vc7 is neither allergenic nor toxic. The instability index is 27.36, which indicates that the vaccine construct is stable under physiological conditions. The aliphatic index has been determined to be 57.84, exceeding the acceptable value of 50 and demonstrating that the vaccine construct is thermally stable across a broad temperature range. The GRAVY score was −0.731, indicating that the vaccine construct is hydrophilic and could interact more effectively with water molecules. The 26.14% of secondary structure of the vaccine is the α-helices, which results in the high binding of antibodies and the formation of H-bonds (Zafar et al., 2022).

The refined vaccine model contained several discontinuous B cell epitopes, indicating its ability to elicit a stronger humoral immune response. The disulfide engineering was performed *in silico* to increase the vaccine’s structural stability. This study identified an unstable residue pair that may be mutated to cysteine to form a disulfide bond (Sinha et al., 2024). The vaccine had low backbone flexibility (S^2^ = 0.74) compared to other designs, thereby ensuring strong interactions with immunological targets, HLA alleles, and TLR4. Vc7 was found to bind with the greatest binding affinity using 2FSE, with a binding energy (−135.24 kcal/mol), and the second highest binding affinity, exhibiting binding energy (−96.18 kcal/mol), was observed against 2Z65. Among the basic requirements for efficient vaccine-receptor interactions, the dynamic stability and rigidity of the complexes must be examined. When the complex is left alone, dynamic stability keeps it intact and correctly aligned throughout molecular motion, and, when appropriately coordinated, it enables the binding interface to remain intact. Vc7-2FSE complex and Vc7-2Z65 complex were both simulated using iMODS. Comparative NMA-based analysis showed that the Vc7-2FSE complex exhibited larger eigenvalues, a higher prevalence of low-frequency modes, better organization of correlated motions, and lower deformability and B-factor, indicating that the complex was more dynamic and structurally more stable than the Vc7-2Z65 complex (Shah et al., 2022).

Structural stability and conformational flexibility of vaccine-receptor complexes were studied using 150 ns MD simulations. The findings revealed that both complexes were conformationally stable and exhibited strong binding, particularly the 2FSE- and 2Z65-vaccine complexes. The PCA and DCCM analyses also supported the stability of the complexes. PCA revealed extensive large-scale motions in the 2FSE and 2Z65 receptors, mainly in the N- and C-terminal regions, and minor reorientation of the vaccine at the binding interface, which together led to greater complex stability. DCCM analysis indicated correlated and anticorrelated movements between the terminal domains of 2FSE and 2Z65, resulting in a compact, energetically favorable conformation upon 2FSE binding. These stability measures are necessary for analyzing protein-protein interactions and are widely used in computational studies.

Once *in silico* vaccine design and validation are completed, the vaccine construct should also be tested for its expressivity and the capacity to elicit host immune response. Optimization of codon and subsequent virtual cloning of the pET-28a (+) plasmid of *E. coli* strain K12 was conducted (Çelik and Çalık, 2012; Rosano and Ceccarelli, 2014). CAI value has been maximized to 1, and GC content has been maximized to 55.30%, which falls within the optimal range of 30%–70% to promote high-level gene expression in the host system. The proposed vaccine construct resulted in a high B cell-mediated immune response as demonstrated by a sharp rise in the B-lymphocyte populations and titers of several immunoglobulins, including combined IgM-IgG, combined IgG1-IgG2, IgM, and IgG subclasses (IgG1 and IgG2), following every dose of injection. This increase in antibody titers is proportional to the decrease in antigen concentration. It triggers various immune mediators, including cytokines and interleukins such as IL-12, IL-10, IL-18, and IL-23, as well as high levels of IFN-γ, the main mediator of host defense against viral infection. This stimulates total B cells and T cells, including Tc cells that destroy infected cells and Th cells that assist in antibody production. It also produces memory T cells, which are vital for prolonged immunity. The proposed vaccine was constructed using immunoinformatics methods in this study, demonstrating significant potential to prevent *Brucella* infection and develop immunity to the pathogen.

Although the present study provides substantial computational evidence supporting the immunogenicity, structural stability, and receptor-binding affinity of the proposed multi-epitope vaccine construct, experimental validation is essential to confirm its biological efficacy and translational applicability. The codon-optimized construct can be cloned into an appropriate *E. coli* expression system for the recombinant expression and purification of the protein under laboratory conditions. Subsequently, *in vitro* immunological assays, including ELISA, cytokine profiling, lymphocyte proliferation assays, western blotting, and flow cytometry, may be employed to evaluate antigenicity and potential for immune activation. In addition, *in vivo* animal model studies can be performed to assess vaccine safety, immunogenicity, bacterial clearance, protective efficacy, and long-term immune memory responses against *Brucella* infection. Such experimental investigations would provide critical validation of the *in silico* predictions and further support the development of the proposed vaccine candidate for future preclinical and clinical studies.

## 5 Conclusion

The *Brucella* spp. remains a significant challenge to human health and the world economy. Humans and livestock may develop a chronic illness due to infection, which may manifest as fatigue, arthritis, and undulant fever. Although brucellosis has a high incidence and severe clinical outcomes, preventive vaccination against the infection has not yet been approved for human use. The toxicity and resistance of current antibacterial drugs commonly limit their efficacy, underscoring the need for a vaccine that is both biologically safe and effective in minimizing morbidity and mortality associated with these drugs. A combined *in silico* bioinformatics strategy incorporating reverse vaccinology and pan-genome approaches was employed to design a universal multi-epitope vaccine against *Brucella* spp. vaccine depending on the contribution of Tig, BamA, UreB, and UreC1 to bacterial pathogenesis and immune evasion. Structural validation, molecular docking, MD simulations, and immune simulation collectively demonstrated the structural stability, favorable receptor-binding affinity, and predicted immunogenic potential of the Vc7 vaccine construct, supported by multiple hydrophobic interactions, hydrogen bonds, and salt bridges with immune receptors. Furthermore, codon optimization and *in silico* cloning analyses supported its potential suitability for heterologous expression. Collectively, these computational validations suggest that Vc7 represents a promising universal multi-epitope vaccine candidate against *Brucella* spp. Nevertheless, additional experimental evidence is needed to assess the therapeutic potential of the Vc7 vaccine candidate targeting *Brucella* spp.

## Data Availability

The original contributions presented in the study are included in the article/Supplementary Material, further inquiries can be directed to the corresponding author.
