# Supplementary material for: An integrated subtractive genomics and immunoinformatics approach for designing a universal multi-epitope vaccine against Brucella spp
Source: Front Bioinform. 2026 Jul 7;6:1818265. doi: 10.3389/fbinf.2026.1818265 (PMC13385411; doi:10.3389/fbinf.2026.1818265)
Supplement: Supplementary file 2 [file Table1.docx]

**Supplementary Table 1:** Subcellular localization of selected proteins.

| **Protein** | **Gneg-Ploc** | **CELLO** | **PSORTb** |
| --- | --- | --- | --- |
| Tig | Periplasm | Cytoplasm | Cytoplasm |
| BamA1 | Outer membrane protein | Outer membrane protein | Outer membrane protein |
| UreB | Periplasm | Cytoplasmic | Cytoplasm |
| UreC1 | Cytoplasm | Cytoplasm | Cytoplasm |
| Der | Cytoplasm | Cytoplasm | Cytoplasm |
