# Supplementary material for: An integrated subtractive genomics and immunoinformatics approach for designing a universal multi-epitope vaccine against Brucella spp
Source: Front Bioinform. 2026 Jul 7;6:1818265. doi: 10.3389/fbinf.2026.1818265 (PMC13385411; doi:10.3389/fbinf.2026.1818265)
Supplement: Supplementary file 3 [file Table8.docx]

**Supplementary Table 8:** Residue-level intermolecular interactions observed between the multi-epitope vaccine construct Vc7 and immune receptor proteins 2FSE and 2Z65.

| **Protein ID** | **Chains** | **Different types of interactions** | **Residue in vaccine** | **Position** | **Vaccine component** |
| --- | --- | --- | --- | --- | --- |
| **2FSE** | **A-U** | **H-bond** | ARG | 86 | Epitope |
|  |  |  | ASN | 112 | Epitope |
|  |  |  | HIS | 176 | His tag |
|  |  | **Salt bridges** | ARG | 86 | Epitope |
|  |  | **Non-bonded contacts** | ARG | 86 | Epitope |
|  |  |  | ASP | 111 | Epitope |
|  |  |  | ASN | 112 | Epitope |
|  |  |  | PRO | 134 | Epitope |
|  |  |  | ALA | 135 | Epitope |
|  |  |  | ASN | 136 | Epitope |
|  |  |  | THR | 137 | Epitope |
|  |  |  | HIS | 172 | His tag |
|  |  |  | HIS | 174 | His tag |
|  |  |  | HIS | 175 | His tag |
|  |  |  | HIS | 176 | His tag |
|  | **B-U** | **H-bond** | ASP | 87 | Epitope |
|  |  |  | ASN | 90 | Epitope |
|  |  |  | ARG | 91 | Epitope |
|  |  |  | GLU | 107 | Epitope |
|  |  |  | ASP | 111 | Epitope |
|  |  |  | ASN | 136 | Epitope |
|  |  |  | LYS | 162 | PADRE |
|  |  |  | HIS | 171 | His tag |
|  |  |  | HIS | 173 | His tag |
|  |  |  | HIS | 174 | His tag |
|  |  | **Salt bridges** | ARG | 91 | Epitope |
|  |  |  | GLU | 107 | Epitope |
|  |  |  | ASP | 111 | Epitope |
|  |  |  | ARG | 140 | Epitope |
|  |  | **Non-bonded contacts** | ASP | 87 | Epitope |
|  |  |  | GLU | 89 | Epitope |
|  |  |  | ASN | 90 | Epitope |
|  |  |  | ARG | 91 | Epitope |
|  |  |  | GLU | 107 | Epitope |
|  |  |  | ASP | 111 | Epitope |
|  |  |  | PRO | 134 | Epitope |
|  |  |  | ASN | 136 | Epitope |
|  |  |  | THR | 137 | Epitope |
|  |  |  | ARG | 140 | Epitope |
|  |  |  | LYS | 162 | PADRE |
|  |  |  | GLY | 170 | PADRE linker |
|  |  |  | HIS | 171 | His tag |
|  |  |  | HIS | 172 | His tag |
|  |  |  | HIS | 173 | His tag |
|  |  |  | HIS | 174 | His tag |
|  |  |  | HIS | 176 | His tag |
|  | **C-U** | **H-bond** | GLN | 12 | Adjuvant |
|  |  |  | TYR | 15 | Adjuvant |
|  |  |  | ARG | 19 | Adjuvant |
|  |  |  | LYS | 31 | Adjuvant |
|  |  |  | GLU | 32 | Adjuvant |
|  |  |  | SER | 39 | Adjuvant |
|  |  | **Salt bridges** | ARG | 19 | Adjuvant |
|  |  |  | GLU | 32 | Adjuvant |
|  |  |  | LYS | 37 | Adjuvant |
|  |  |  | ARG | 41 | Adjuvant |
|  |  |  | LYS | 70 | PADRE |
|  |  | **Non-bonded contacts** | GLU | 1 | Adjuvant linker |
|  |  |  | LYS | 5 | Adjuvant linker |
|  |  |  | GLN | 12 | Adjuvant |
|  |  |  | TYR | 15 | Adjuvant |
|  |  |  | CYS | 16 | Adjuvant |
|  |  |  | ARG | 19 | Adjuvant |
|  |  |  | LYS | 31 | Adjuvant |
|  |  |  | GLU | 32 | Adjuvant |
|  |  |  | ILE | 35 | Adjuvant |
|  |  |  | GLY | 36 | Adjuvant |
|  |  |  | LYS | 37 | Adjuvant |
|  |  |  | CYS | 38 | Adjuvant |
|  |  |  | SER | 39 | Adjuvant |
|  |  |  | THR | 40 | Adjuvant |
|  |  |  | ARG | 41 | Adjuvant |
|  |  |  | LYS | 70 | PADRE linker |
| **2Z65** | **A-U** | **H-bonds** | ARG | 97 | Epitopic linker |
|  |  |  | ARG | 98 | Epitopic linker |
|  |  |  | GLU | 120 | Epitope |
|  |  |  | PHE | 121 | Epitope |
|  |  |  | HIS | 174 | His tag |
|  |  | **Salt bridges** | ARG | 98 | Epitopic linker |
|  |  |  | LYS | 100 | Epitope |
|  |  |  | GLU | 120 | Epitope |
|  |  |  | GLU | 146 | Epitope |
|  |  |  | HIS | 172 | His tag |
|  |  | **Non-bonded contacts** | ALA | 2 | Adjuvant linker |
|  |  |  | PRO | 57 | PADRE linker |
|  |  |  | ARG | 97 | Epitopic linker |
|  |  |  | ARG | 98 | Epitopic linker |
|  |  |  | GLY | 99 | Epitope |
|  |  |  | LYS | 100 | Epitope |
|  |  |  | ASP | 102 | Epitope |
|  |  |  | GLU | 120 | Epitope |
|  |  |  | PHE | 121 | Epitope |
|  |  |  | ARG | 123 | Epitope |
|  |  |  | ASN | 136 | Epitope |
|  |  |  | GLU | 146 | Epitope |
|  |  |  | PRO | 151 | PADRE linker |
|  |  |  | GLY | 152 | PADRE linker |
|  |  |  | ALA | 165 | PADRE |
|  |  |  | PRO | 167 | PADRE linker |
|  |  |  | GLY | 168 | PADRE linker |
|  |  |  | PRO | 169 | PADRE linker |
|  |  |  | HIS | 171 | His tag |
|  |  |  | HIS | 172 | His tag |
|  |  |  | HIS | 174 | His tag |
|  |  |  | HIS | 175 | His tag |
|  |  |  | HIS | 176 | His tag |
