# Supplementary material for: An integrated subtractive genomics and immunoinformatics approach for designing a universal multi-epitope vaccine against Brucella spp
Source: Front Bioinform. 2026 Jul 7;6:1818265. doi: 10.3389/fbinf.2026.1818265 (PMC13385411; doi:10.3389/fbinf.2026.1818265)
Supplement: Supplementary file 6 [file Table7.docx]

**Supplementary Table 7:** List of residue pairs in the vaccine construct predicted to form disulfide bonds, accompanied by their energy scores.

| **Res1 AA** | **Res2 AA** | **Energy (kcal/mol)** |
| --- | --- | --- |
| ALA | GLY | 3.58 |
| ILE | GLY | 2.52 |
| ALA | GLY | 2.28 |
| VAL | CYS | 5.26 |
| LEU | ALA | 11.41 |
| LEU | TRP | 3.42 |
| CYS | PHE | 3.36 |
| ALA | GLY | 5.86 |
| ALA | ALA | 4.01 |
| PRO | GLY | 4.01 |
| LYS | SER | 6 |
| ALA | LYS | 6.83 |
| ALA | PRO | 2.71 |
| SER | LEU | 4.19 |
| ARG | ALA | 9.89 |
| ARG | LYS | 5.9 |
| PHE | ARG | 7.23 |
| GLY | ALA | 7.97 |
| GLU | ASP | 3.43 |
| GLU | LYS | 6.7 |
| LEU | ALA | 4.97 |
