# Supplementary material for: An integrated subtractive genomics and immunoinformatics approach for designing a universal multi-epitope vaccine against Brucella spp
Source: Front Bioinform. 2026 Jul 7;6:1818265. doi: 10.3389/fbinf.2026.1818265 (PMC13385411; doi:10.3389/fbinf.2026.1818265)
Supplement: Supplementary file 9 [file Table2.docx]

**Supplementary Table 2:** Assessment of 46 epitopes of MHC-I T-lymphocytes, showcasing scores for immunogenicity, antigenicity, and toxicity.

| **Protein Name** | **NCBI Accession Number** | **Predicted Epitope Sequence** | **Epitope Position** | **Immunogenicity** | **Antigenicity** | **Toxicity** |
| --- | --- | --- | --- | --- | --- | --- |
| Tig | CDL76300.1 | SSTRTFETK | 156-164 | 0.31986 | 1.3333 | Non-Toxin |
|  |  | EAAREEYRK | 345-353 | 0.28365 | 1.1338 | Non-Toxin |
|  |  | KLAERLETA | 29-37 | 0.26059 | 0.6788 | Non-Toxin |
|  |  | EVLPAIEVK | 120-128 | 0.25724 | 0.8114 | Non-Toxin |
|  |  | FIPGFEEQL | 204-212 | 0.25557 | 0.7721 | Non-Toxin |
|  |  | KVITVTFPA | 221-229 | 0.25557 | 0.6506 | Non-Toxin |
|  |  | RKLAERRVR | 352-360 | 0.24979 | 1.2267 | Non-Toxin |
|  |  | ITVTFPAEY | 223-231 | 0.24754 | 0.6602 | Non-Toxin |
|  |  | LVDAEFNNI | 314-322 | 0.24503 | 0.6902 | Non-Toxin |
|  |  | AENEDRVTI | 168-176 | 0.22649 | 0.8849 | Non-Toxin |
|  |  | ETARGRARI | 35-43 | 0.20976 | 0.8153 | Non-Toxin |
|  |  | YEVLPAIEV | 119-127 | 0.19909 | 0.5458 | Non-Toxin |
|  |  | DEKVITVTF | 219-227 | 0.1952 | 0.9747 | Non-Toxin |
| BamA | GAA5662309.1 | FVIRREFDV | 376-384 | 0.35169 | 1.0678 | Non-Toxin |
|  |  | NEYTITITV | 264-272 | 0.339 | 1.0441 | Non-Toxin |
|  |  | KTRDFVIRR | 372-380 | 0.33454 | 0.6505 | Non-Toxin |
|  |  | SLGIRGAVF | 708-716 | 0.28436 | 1.1729 | Non-Toxin |
|  |  | MEADVEAIK | 142-150 | 0.28025 | 1.2405 | Non-Toxin |
|  |  | FDYAFPIAK | 760-768 | 0.27691 | 0.704 | Non-Toxin |
|  |  | STNEYTITI | 262-270 | 0.26659 | 1.5476 | Non-Toxin |
|  |  | ESLGIRGAV | 707-715 | 0.26028 | 1.3369 | Non-Toxin |
|  |  | TSADIDAAV | 70-78 | 0.24138 | 1.0797 | Non-Toxin |
|  |  | DEETLRRFY | 234-242 | 0.22156 | 0.7832 | Non-Toxin |
|  |  | RRLEALDFF | 400-408 | 0.21193 | 0.3882 | Non-Toxin |
|  |  | FSAGIAYNL | 535-543 | 0.20633 | 1.2075 | Non-Toxin |
|  |  | GPLRFDYAF | 756-764 | 0.2031 | 0.9814 | Non-Toxin |
|  |  | TYINGTAEV | 690-698 | 0.19775 | 0.4154 | Non-Toxin |
|  |  | ATMEADVEA | 140-148 | 0.19607 | 1.4972 | Non-Toxin |
|  |  | LRFDYAFPI | 758-766 | 0.18587 | 2.452 | Non-Toxin |
|  |  | KVQNFNFGV | 773-781 | 0.1824 | 1.4262 | Non-Toxin |
|  |  | FTSADIDAA | 69-77 | 0.17413 | 0.6980 | Non-Toxin |
|  |  | RLSAGFDVF | 498-506 | 0.17171 | 0.9652 | Non-Toxin |
|  |  | AKEIEDSVL | 311-319 | 0.1693 | 0.4008 | Non-Toxin |
|  |  | EPYFLGYRL | 491-499 | 0.16482 | 0.7973 | Non-Toxin |
|  |  | GQGRVNVVY | 171-179 | 0.15615 | 0.6856 | Non-Toxin |
|  |  | EFGDDGVRI | 645-653 | 0.1519 | 0.5861 | Non-Toxin |
| UreB | AHN46974.1 | RFIFGFNNL | 111-119 | 0.29496 | 0.6925 | Non-Toxin |
|  |  | EIAAERAEK | 137-145 | 0.28981 | 1.1457 | Non-Toxin |
|  |  | REIAAERAE | 136-144 | 0.28146 | 0.7359 | Non-Toxin |
|  |  | YQPNREIAA | 132-140 | 0.26972 | 0.4587 | Non-Toxin |
|  |  | FIFGFNNLV | 112-120 | 0.16807 | 0.8792 | Non-Toxin |
| UreC1 | SUW39063.1 | PAIPEDIAF | 326-334 | 0.2856 | 0.4107 | Non-Toxin |
|  |  | LWNPAFFGV | 436-444 | 0.25364 | 0.9894 | Non-Toxin |
|  |  | YEVRADGEL | 544-552 | 0.21156 | 1.5161 | Non-Toxin |
|  |  | TEGAGGGHA | 275-283 | 0.16277 | 3.2075 | Non-Toxin |
|  |  | QAMGRVGEM | 364-372 | 0.15346 | 0.2118 | Non-Toxin |
