# Supplementary material for: An integrated subtractive genomics and immunoinformatics approach for designing a universal multi-epitope vaccine against Brucella spp
Source: Front Bioinform. 2026 Jul 7;6:1818265. doi: 10.3389/fbinf.2026.1818265 (PMC13385411; doi:10.3389/fbinf.2026.1818265)
Supplement: Supplementary file 10 [file Table5.docx]

**Supplementary Table 5:** Quality parameters of alternative configurations of vaccine constructs.

| **Properties** | **VC1** | **VC3** | **VC4** | **VC5** | **VC6** | **VC7** | **VC8** | **VC9** | **VC11** |
| --- | --- | --- | --- | --- | --- | --- | --- | --- | --- |
| Quality Factor | 95.4545 | 85.8065 | 89.7059 | 88.1226 | 93.7759 | 91.9255 | 93.3673 | 83.3977 | 89.172 |
| Ramachandran Favored | 95.50% | 89.08% | 96.14% | 97.57% | 94.27% | 95.98% | 96.14% | 95.14% | 93.68% |
| Verify3D Structural Violations | Fail | Fail | Fail | Fail | Fail | Pass | Fail | Fail | Pass |
| Distribution Z- Score | 1.79 ± 0.44 | -1.71 ± 0.57 | -0.40 ± 0.47 | 1.50 ± 0.45 | 1.59 ± 0.45 | -0.44 ± 0.56 | -0.37 ± 0.49 | 1.93 ± 0.47 | -0.43 ± 0.59 |
| Backbone Dynamics (Average S^2^-parameter scores) | 0.757565 | 0.743292 | 0.76913 | 0.758827 | 0.752541 | 0.743292 | 0.76913 | 0.758827 | 0.743292 |
| LGScore | 6.752 | 6.838 | 11.516 | 9.276 | 9.004 | 5.757 | 11.459 | 7.068 | 5.003 |
| MaxSub | -0.663 | -0.556 | -1.061 | -0.743 | -0.688 | -0.601 | -0.875 | -0.589 | -0.559 |
