# Supplementary material for: An integrated subtractive genomics and immunoinformatics approach for designing a universal multi-epitope vaccine against Brucella spp
Source: Front Bioinform. 2026 Jul 7;6:1818265. doi: 10.3389/fbinf.2026.1818265 (PMC13385411; doi:10.3389/fbinf.2026.1818265)
Supplement: Supplementary file 11 [file Table3.docx]

**Supplementary Table 3:** Shortlisted 51 final CD4+ T-cell epitopes with their immunogenicity, antigenicity, and toxicity scores.

| **Protein Name** | **NCBI Accession Number** | **Predicted Epitope Sequence** | **Epitope Position** | **Immunogenicity** | **Antigenicity** | **Toxicity** |
| --- | --- | --- | --- | --- | --- | --- |
| Tig | CDL76300.1 | AREEYRKLAERRVRL | 347-361 | 84.4261 | 0.8895 | Non-Toxin |
|  |  | DGKADFVFSLNYEVL | 108-122 | 92.979 | 1.3188 | Non-Toxin |
|  |  | GKADFVFSLNYEVLP | 109-123 | 92.3544 | 1.1663 | Non-Toxin |
|  |  | KADFVFSLNYEVLPA | 110-124 | 92.2693 | 1.0702 | Non-Toxin |
|  |  | ADFVFSLNYEVLPAI | 111-125 | 94.0213 | 0.9981 | Non-Toxin |
|  |  | LDGKADFVFSLNYEV | 107-121 | 97.5049 | 1.4453 | Non-Toxin |
|  |  | DEQVKRIASSTRTFE | 148-162 | 92.1658 | 0.4945 | Non-Toxin |
|  |  | FVFSLNYEVLPAIEV | 113-127 | 96.5779 | 1.3067 | Non-Toxin |
| BamA | GAA5662309.1 | FGPLRFDYAFPIAKA | 755-769 | 63.5415 | 0.5683 | Non-Toxin |
|  |  | PFGPLRFDYAFPIAK | 754-768 | 65.8833 | 0.4318 | Non-Toxin |
|  |  | GPLRFDYAFPIAKAD | 756-770 | 70.2638 | 0.7785 | Non-Toxin |
|  |  | SPFGPLRFDYAFPIA | 753-767 | 68.2552 | 0.6027 | Non-Toxin |
|  |  | PLRFDYAFPIAKADT | 757-771 | 71.0248 | 1.2262 | Non-Toxin |
|  |  | ASPFGPLRFDYAFPI | 752-766 | 69.3876 | 0.6618 | Non-Toxin |
|  |  | LGYRLSAGFDVFRRT | 495-509 | 74.6891 | 0.4973 | Non-Toxin |
|  |  | LRFDYAFPIAKADTD | 758-772 | 71.1939 | 1.306 | Non-Toxin |
|  |  | GYADFRVISSNAVLD | 246-260 | 87.5585 | 0.6326 | Non-Toxin |
|  |  | YFLGYRLSAGFDVFR | 493-507 | 80.28 | 0.5357 | Non-Toxin |
|  |  | YADFRVISSNAVLDP | 247-261 | 87.7204 | 0.6578 | Non-Toxin |
|  |  | RGYADFRVISSNAVL | 245-259 | 87.6849 | 0.4949 | Non-Toxin |
|  |  | PYFLGYRLSAGFDVF | 492-506 | 81.9302 | 0.9114 | Non-Toxin |
|  |  | NRGYADFRVISSNAV | 244-258 | 88.04 | 0.5621 | Non-Toxin |
|  |  | ADFRVISSNAVLDPS | 248-262 | 89.7048 | 0.7801 | Non-Toxin |
|  |  | DFRVISSNAVLDPST | 249-262 | 91.4791 | 0.7466 | Non-Toxin |
|  |  | EPYFLGYRLSAGFDV | 491-505 | 84.7276 | 1.0561 | Non-Toxin |
|  |  | FRVISSNAVLDPSTN | 250-264 | 93.6501 | 0.4215 | Non-Toxin |
|  |  | ADIDAAVKRLFAMGL | 72-86 | 91.416 | 0.6328 | Non-Toxin |
|  |  | DIDAAVKRLFAMGLF | 73-87 | 93.6078 | 0.4373 | Non-Toxin |
|  |  | LRSSISYSLTYNSID | 573-587 | 86.071 | 0.5715 | Non-Toxin |
|  |  | VDFVGNQAFSSRRLR | 192-206 | 95.5062 | 0.4027 | Non-Toxin |
|  |  | LGRGQYIRISAGAGQ | 465-479 | 89.5929 | 1.1130 | Non-Toxin |
|  |  | LFSDVRINQSGSTLV | 86-100 | 95.8863 | 0.6404 | Non-Toxin |
|  |  | MSVALVASGTAALSL | 16-30 | 96.7266 | 0.5088 | Non-Toxin |
|  |  | FLGRGQYIRISAGAG | 464-478 | 86.306 | 0.7063 | Non-Toxin |
|  |  | TDKVQNFNFGVSTKF | 771-785 | 94.4602 | 0.7772 | Non-Toxin |
|  |  | FSDVRINQSGSTLVV | 87-101 | 97.5148 | 0.9159 | Non-Toxin |
|  |  | SDVRINQSGSTLVVN | 88-102 | 98.3721 | 1.2922 | Non-Toxin |
|  |  | GRGQYIRISAGAGQD | 466-480 | 87.3677 | 1.6627 | Non-Toxin |
|  |  | DVRINQSGSTLVVNV | 89-103 | 98.2192 | 1.5458 | Non-Toxin |
|  |  | RGQYIRISAGAGQDD | 467-481 | 84.8088 | 1.3362 | Non-Toxin |
|  |  | GQYIRISAGAGQDDM | 468-482 | 83.8153 | 1.1286 | Non-Toxin |
|  |  | DMRNYGLSFTEPYFL | 481-495 | 91.5637 | 0.6274 | Non-Toxin |
|  |  | VRINQSGSTLVVNVT | 90-104 | 97.6045 | 0.9856 | Non-Toxin |
|  |  | IANVDFVGNQAFSSR | 189-203 | 96.5668 | 0.4385 | Non-Toxin |
|  |  | SALAMSVALVASGTA | 12--26 | 93.7234 | 0.4436 | Non-Toxin |
| UreB | AHN46974.1 | LEFDRSKAFGLRLDI | 72-86 | 90.7803 | 1.0524 | Non-Toxin |
|  |  | AGKRFIFGFNNLVDG | 108-122 | 81.4527 | 0.4662 | Non-Toxin |
| UreC1 | SUW39063.1 | GKHSMILNNAMPQME | 524-538 | 60.133 | 0.4583 | Non-Toxin |
|  |  | KHSMILNNAMPQMEV | 525-539 | 63.5734 | 0.4045 | Non-Toxin |
|  |  | GIGKHSMILNNAMPQ | 522-536 | 62.1155 | 0.4331 | Non-Toxin |
|  |  | HSMILNNAMPQMEVD | 526-540 | 78.2913 | 0.6601 | Non-Toxin |
