# Supplementary material for: An integrated subtractive genomics and immunoinformatics approach for designing a universal multi-epitope vaccine against Brucella spp
Source: Front Bioinform. 2026 Jul 7;6:1818265. doi: 10.3389/fbinf.2026.1818265 (PMC13385411; doi:10.3389/fbinf.2026.1818265)
Supplement: Supplementary file 12 [file Table4.docx]

**Supplementary Table 4:** Alternative configurations of vaccine constructs.

| Vc1 | HBHA, E1, E2, E3, linkers, PADRE, His Tag | EAAAKMAENPNIDDLPAPLLAALGAADLALATVNDLIANLRERAEETRAETRTRVEERRARLTKFQEDLPEQFIELRDKFTTEELRKAAEGYLEAATNRYNELVERGEAALQRLRSQTAFEDASARAEGYVDQAVELTQEALGTVASQTRAVGERAAKLVGIELEAAAKGPGPGAKFVAAWTLKAAAGPGPGGKLDGEPFEGGADNDARVRRKVEPRGDRDFENRTISRVRRLEFDRSKAFGLRLDIPANTAVRFEPGDEKGPGPGAKFVAAWTLKAAAGPGPGHHHHHHH |
| --- | --- | --- |
| Vc2 | HBHA conserved, E1, E2, E3, linkers, PADRE, His Tag | EAAAKMAENSNIDDIKAPLLAALGAADLALATVNELITNLRERAEETRRSRVEESRARLTKLQEDLPEQLTELREKFTAEELRKAAEGYLEAATSELVERGEAALERLRSQQSFEEVSARAEGYVDQAVELTQEALGTVASQVEGRAAKLVGIELEAAAKGPGPGAKFVAAWTLKAAAGPGPGGKLDGEPFEGGADNDARVRRKVEPRGDRDFENRTISRVRRLEFDRSKAFGLRLDIPANTAVRFEPGDEKGPGPGAKFVAAWTLKAAAGPGPGHHHHHH |
| Vc3 | B-Defensin, E1, E2, E3, linkers, PADRE, His Tag | EAAAKGIINTLQKYYCRVRGGRCAVLSCLPKEEQIGKCSTRGRKCCRRKKEAAAKGPGPGAKFVAAWTLKAAAGPGPGGKLDGEPFEGGADNDARVRRKVEPRGDRDFENRTISRVRRLEFDRSKAFGLRLDIPANTAVRFEPGDEKGPGPGAKFVAAWTLKAAAGPGPGHHHHHH |
| Vc4 | L7/L12 Ribosomal protein, E1, E2, E3, linkers, PADRE, His Tag | EAAAKMAKLSTDELLDAFKEMTLLELSDFVKKFEETFEVTAAAPVAVAAAGAAPAGAAVEAAEEQSEFDVILEAAGDKKIGVIKVVREIVSGLGLKEAKDLVDGAPKPLLEKVAKEAADEAKAKLEAAGATVTVKEAAAKGPGPGAKFVAAWTLKAAAGPGPGGKLDGEPFEGGADNDARVRRKVEPRGDRDFENRTISRVRRLEFDRSKAFGLRLDIPANTAVRFEPGDEKGPGPGAKFVAAWTLKAAAGPGPGHHHHHH |
| Vc5 | HBHA, E2, E1, E3, linkers, PADRE, His Tag | EAAAKMAENPNIDDLPAPLLAALGAADLALATVNDLIANLRERAEETRAETRTRVEERRARLTKFQEDLPEQFIELRDKFTTEELRKAAEGYLEAATNRYNELVERGEAALQRLRSQTAFEDASARAEGYVDQAVELTQEALGTVASQTRAVGERAAKLVGIELEAAAKGPGPGAKFVAAWTLKAAAGPGPGKVEPRGDRDFENRTISRVRRGKLDGEPFEGGADNDARVRRLEFDRSKAFGLRLDIPANTAVRFEPGDEKGPGPGAKFVAAWTLKAAAGPGPGHHHHHH |
| Vc6 | HBHA conserved, E2, E1, E3, linkers, PADRE, His Tag | EAAAKMAENSNIDDIKAPLLAALGAADLALATVNELITNLRERAEETRRSRVEESRARLTKLQEDLPEQLTELREKFTAEELRKAAEGYLEAATSELVERGEAALERLRSQQSFEEVSARAEGYVDQAVELTQEALGTVASQVEGRAAKLVGIELEAAAKGPGPGAKFVAAWTLKAAAGPGPGKVEPRGDRDFENRTISRVRRGKLDGEPFEGGADNDARVRRLEFDRSKAFGLRLDIPANTAVRFEPGDEKGPGPGAKFVAAWTLKAAAGPGPGHHHHHH |
| Vc7 | B-Defensin, E2, E1, E3, linkers, PADRE, His Tag | EAAAKGIINTLQKYYCRVRGGRCAVLSCLPKEEQIGKCSTRGRKCCRRKKEAAAKGPGPGAKFVAAWTLKAAAGPGPGKVEPRGDRDFENRTISRVRRGKLDGEPFEGGADNDARVRRLEFDRSKAFGLRLDIPANTAVRFEPGDEKGPGPGAKFVAAWTLKAAAGPGPGHHHHHH |
| Vc8 | L7/L12 Ribosomal protein, E2, E1, E3, linkers, PADRE, His Tag | EAAAKMAKLSTDELLDAFKEMTLLELSDFVKKFEETFEVTAAAPVAVAAAGAAPAGAAVEAAEEQSEFDVILEAAGDKKIGVIKVVREIVSGLGLKEAKDLVDGAPKPLLEKVAKEAADEAKAKLEAAGATVTVKEAAAKGPGPGAKFVAAWTLKAAAGPGPGKVEPRGDRDFENRTISRVRRGKLDGEPFEGGADNDARVRRLEFDRSKAFGLRLDIPANTAVRFEPGDEKGPGPGAKFVAAWTLKAAAGPGPGHHHHHH |
| Vc9 | HBHA, E3, E2, E1, linkers, PADRE, His Tag | EAAAKMAENPNIDDLPAPLLAALGAADLALATVNDLIANLRERAEETRAETRTRVEERRARLTKFQEDLPEQFIELRDKFTTEELRKAAEGYLEAATNRYNELVERGEAALQRLRSQTAFEDASARAEGYVDQAVELTQEALGTVASQTRAVGERAAKLVGIELEAAAKGPGPGAKFVAAWTLKAAAGPGPGLEFDRSKAFGLRLDIPANTAVRFEPGDEKRVRRKVEPRGDRDFENRTISRVRRGKLDGEPFEGGADNDAGPGPGAKFVAAWTLKAAAGPGPGHHHHHH |
| Vc10 | HBHA conserved, E3, E2, E1, linkers, PADRE, His Tag | EAAAKMAENSNIDDIKAPLLAALGAADLALATVNELITNLRERAEETRRSRVEESRARLTKLQEDLPEQLTELREKFTAEELRKAAEGYLEAATSELVERGEAALERLRSQQSFEEVSARAEGYVDQAVELTQEALGTVASQVEGRAAKLVGIELEAAAKGPGPGAKFVAAWTLKAAAGPGPGLEFDRSKAFGLRLDIPANTAVRFEPGDEKRVRRKVEPRGDRDFENRTISRVRRGKLDGEPFEGGADNDAGPGPGAKFVAAWTLKAAAGPGPGHHHHHH |
| Vc11 | B-Defensin, E3, E2, E1, linkers, PADRE, His Tag | EAAAKGIINTLQKYYCRVRGGRCAVLSCLPKEEQIGKCSTRGRKCCRRKKEAAAKGPGPGAKFVAAWTLKAAAGPGPGLEFDRSKAFGLRLDIPANTAVRFEPGDEKRVRRKVEPRGDRDFENRTISRVRRGKLDGEPFEGGADNDAGPGPGAKFVAAWTLKAAAGPGPGHHHHHH |
| Vc12 | L7/L12 Ribosomal protein, E3, E2, E1, linkers, PADRE, His Tag | EAAAKMAKLSTDELLDAFKEMTLLELSDFVKKFEETFEVTAAAPVAVAAAGAAPAGAAVEAAEEQSEFDVILEAAGDKKIGVIKVVREIVSGLGLKEAKDLVDGAPKPLLEKVAKEAADEAKAKLEAAGATVTVKEAAAKGPGPGAKFVAAWTLKAAAGPGPGLEFDRSKAFGLRLDIPANTAVRFEPGDEKRVRRKVEPRGDRDFENRTISRVRRGKLDGEPFEGGADNDAGPGPGAKFVAAWTLKAAAGPGPGHHHHHH |

*Colour code legend: Orange coloured amino acids - linker to connect adjuvants, black coloured amino acid - adjuvants, pink coloured amino acids - PADRE sequence linker, sky-blue coloured amino acids- PADRE sequence, green coloured amino acid - epitope A, purple coloured amino acid - epitopic linker, grey coloured amino acids - epitope B, red coloured amino acids - epitope C, brown coloured amino acids - His-tag*

.
