# Supplementary material for: An integrated subtractive genomics and immunoinformatics approach for designing a universal multi-epitope vaccine against Brucella spp
Source: Front Bioinform. 2026 Jul 7;6:1818265. doi: 10.3389/fbinf.2026.1818265 (PMC13385411; doi:10.3389/fbinf.2026.1818265)
Supplement: Supplementary file 13 [file Table6.docx]

**Supplementary Table 6:** Predicted discontinuous B-cell epitope clusters with residue positions and confidence scores.

| **No.** | **Residues** | **Number of residues** | **Score** |
| --- | --- | --- | --- |
| 1 | A:L161, A:K162, A:A163, A:A164, A:A165, A:G166, A:P167, A:G168, A:P169, A:G170, A:H171 | 11 | 0.709 |
| 2 | A:C16, A:V18, A:R19, A:G20, A:G21, A:R22, A:C23, A:A24, A:I35, A:G36, A:K37, A:C38, A:S39, A:T40, A:R41, A:G42, A:R43, A:K44, A:C45, A:R47, A:R48, A:A73, A:G74, A:P75, A:G76, A:P77, A:G78, A:K79, A:V80, A:E81, A:P82, A:G84, A:D85, A:N90 | 34 | 0.707 |
| 3 | A:R98, A:G99, A:K100, A:L101, A:D102, A:G103, A:E104, A:P105, A:F106, A:E107, A:G108, A:G109, A:A110, A:D111, A:N112, A:A114, A:R115, A:E120, A:I133, A:P134, A:A135, A:N136, A:T137, A:A138, A:V139, A:R140, A:F141, A:E142, A:P143, A:G144, A:D145, A:E146, A:K147, A:G148, A:P149, A:A153, A:K154, A:F155, A:H172, A:H173, A:H174, A:H175, A:H176 | 43 | 0.63 |
| 4 | A:E51, A:A52, A:A54, A:K55, A:G56, A:P57, A:G58, A:G150, A:P151, A:G152 | 10 | 0.576 |
| 5 | A:R86, A:D87, A:F88, A:D132 | 4 | 0.506 |
