# Supplementary figures and images for: An integrated subtractive genomics and immunoinformatics approach for designing a universal multi-epitope vaccine against Brucella spp
Source: Front Bioinform. 2026 Jul 7;6:1818265. doi: 10.3389/fbinf.2026.1818265 (PMC13385411; doi:10.3389/fbinf.2026.1818265)

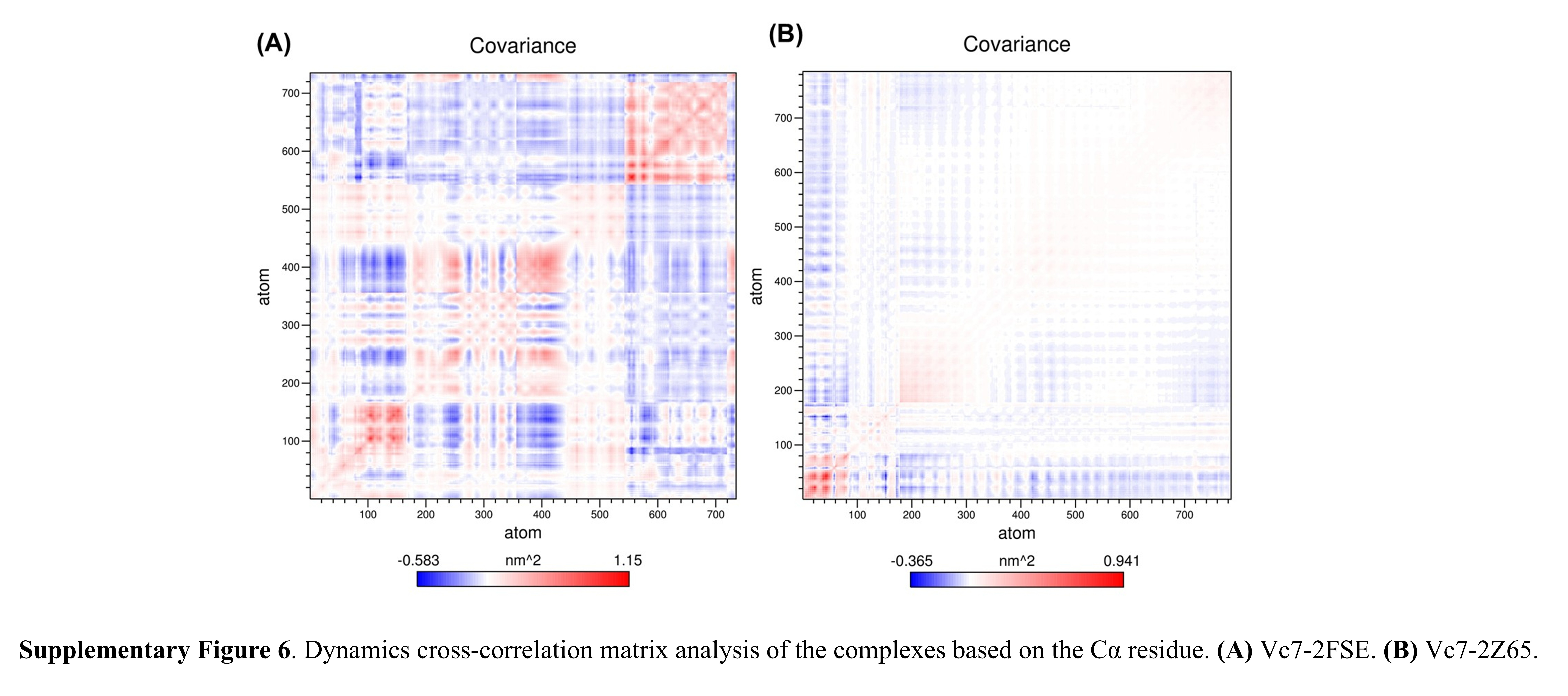

Supplement: Supplementary file 14 [file Image6.jpeg]
